# Photobinding of Triflusal to Human Serum Albumin Investigated by Fluorescence, Proteomic Analysis, and Computational Studies

**DOI:** 10.3389/fphar.2019.01028

**Published:** 2019-09-20

**Authors:** Oscar Molins-Molina, Raúl Pérez-Ruiz, Emilio Lence, Concepción González-Bello, Miguel A. Miranda, M. Consuelo Jiménez

**Affiliations:** ^1^Departamento de Química/Instituto de Tecnología Química UPV-CSIC, Universitat Politécnica de València, Valencia, Spain; ^2^Centro Singular de Investigación en Química Biolóxica e Materiais Moleculares (CiQUS), Departamento de Química Orgánica, Universidade de Santiago de Compostela, Santiago de Compostela, Spain

**Keywords:** triflusal metabolite, human serum albumin, fluorescence, proteomic analysis, docking and molecular dynamics

## Abstract

Triflusal is a platelet antiaggregant employed for the treatment and prevention of thromboembolic diseases. After administration, it is biotransformed into its active metabolite, the 2-hydroxy-4-trifluoromethylbenzoic acid (HTB). We present here an investigation on HTB photobinding to human serum albumin (HSA), the most abundant protein in plasma, using an approach that combines fluorescence, MS/MS, and peptide fingerprint analysis as well as theoretical calculations (docking and molecular dynamics simulation studies). The proteomic analysis of HTB/HSA photolysates shows that HTB addition takes place at the ε-amino groups of the Lys137, Lys199, Lys205, Lys351, Lys432, Lys525, Lys541 and Lys545 residues and involves replacement of the trifluoromethyl moiety of HTB with a new amide function. Only Lys199 is located in an internal pocket of the protein, and the remaining modified residues are placed in the external part. Docking and molecular dynamic simulation studies reveal that HTB supramolecular binding to HSA occurs in the “V-cleft” region and that the process is assisted by the presence of Glu/Asp residues in the neighborhood of the external Lys, in agreement with the experimentally observed modifications. In principle, photobinding can occur with other trifluoroaromatic compounds and may be responsible for the appearance of undesired photoallergic side effects.

## Introduction

Photoactive molecules can be present in living systems as endogenous substances or they can be taken up from exogenous sources (Epstein, 1983; Stein and Scheinfeld, 2007; Onoue et al., 2009). They include drugs, cosmetics, pesticides, or dyes and can produce beneficial effects in living organisms, which can be used for therapeutic purposes; however, they can be turned into a biological damaging agent by non-harmful and low energetic light, triggering a cascade of chemical events that may finally result in important biological disorders (Epstein and Wintroub, 1985; Reavy et al., 1997; Moore, 1998; Sauvaigo et al., 2001; Moore, 2002; Cuquerella et al., 2012).

In this context, photoallergy is associated with a cell-mediated immune response which is initiated by covalent binding of a light-activated hapten (for instance, a drug or a species derived therefrom) to a protein (Tokura, 2009; Ariza et al., 2011; Onoue et al., 2017). It is considered an emerging health concern due to the widespread use of topical drugs (including antibiotics, antifungals, antihistaminics, cardiovascular and nonsteroidal anti-inflammatory drugs), cosmetics, and nutraceutical in humans, which has attracted considerable attention from both industry and regulatory agencies (Schothorst et al., 1972; Girotti, 2001; Deleo, 2004; Dubakiene and Kupriene, 2006; Scheuer and Warshaw, 2006; Lugovic et al., 2007; Bylaite et al., 2009; Santoro and Lim, 2011; Elkeeb et al., 2012; Onoue et al., 2013; Honari, 2014; Kerstein et al., 2014; Scheinfeld et al., 2014; Onoue et al., 2016).

Triflusal (2-acetoxy-4-trifluoromethylbenzoic acid), a platelet antiaggregant, is employed for the treatment and prevention of thromboembolic diseases (Messa et al., 1993; Plaza et al., 1993; McNeely and Goa, 1998; Gonzalez-Correa and De La Cruz, 2006). In fact, it acts as prodrug that after administration is biotransformed into its active metabolite, the 2-hydroxy-4-trifluoromethylbenzoic acid (HTB), whose half-life in the organism is 70-fold longer than that of triflusal. It has been demonstrated that not only triflusal but also HTB is capable to induce photoallergy in humans (Serrano et al., 1987; Lee et al., 1999; Nagore et al., 2000; Lee et al., 2001). In this context, HTB has been found to be photolabile under various conditions. Its major photodegradation pathway appears to be the nucleophilic attack at the trifluoromethyl moiety. In the presence of nucleophiles (including amino acids, peptides, or proteins), carboxylic acid derivatives (esters, amides, thioesters) are formed.

Photobinding of HTB to bovine serum albumin has been previously monitored in our group through the fluorescence changes occurring upon irradiation of a drug–protein mixture, after isolation of the protein by gel-filtration chromatography (Boscá et al., 2001). In addition, formation of photoadducts between HTB and lysine or polylysine (Montanaro et al., 2009) as well as HTB photoreaction with lysine residues of the simple model protein ubiquitin (Nuin et al., 2016) have been observed. This points to a photonucleophilic addition of the lysine amino group to HTB as a key step in photoallergy. For a simplified reaction mechanism, see **Scheme S1** in the ESI.

However, ubiquitin is a small protein that lacks binding sites and does not bind to drugs efficiently; therefore, it appears necessary to employ as target a transport protein present in human blood that is able to interact with HTB at some stage while the drug is developing its pharmacological action. This is the case of human serum albumin (HSA), the most abundant protein in human plasma, which is able to bind a widespread range of endo- and exogeneous ligands. It is a 67-kDa monomer whose primary structure comprises a single chain of 585 amino acid residues, with 17 disulfide bridges, 1 tryptophan, and 1 free cysteine; a α-helix of six turns forms the 67% of the secondary structure. The 3D assembly of HSA contains three homologous helical domains (I-III), each divided into A and B subdomains (Peters, 1995; Fasano et al., 2005; Ghuman et al., 2005). Regarding the seminal work of Sudlow and co-workers based on the displacement of fluorescence probes, small ligands usually bind at one of the two primary sites (I and II) located in subdomains IIA and IIIA, respectively. Although to a lesser extent, sites with lower affinity can also be populated. The binding constant of HTB to HSA is 4.7×10^5^ M^−1^, with site I as the main binding site (Mis et al., 1992).

**Figure 1:**
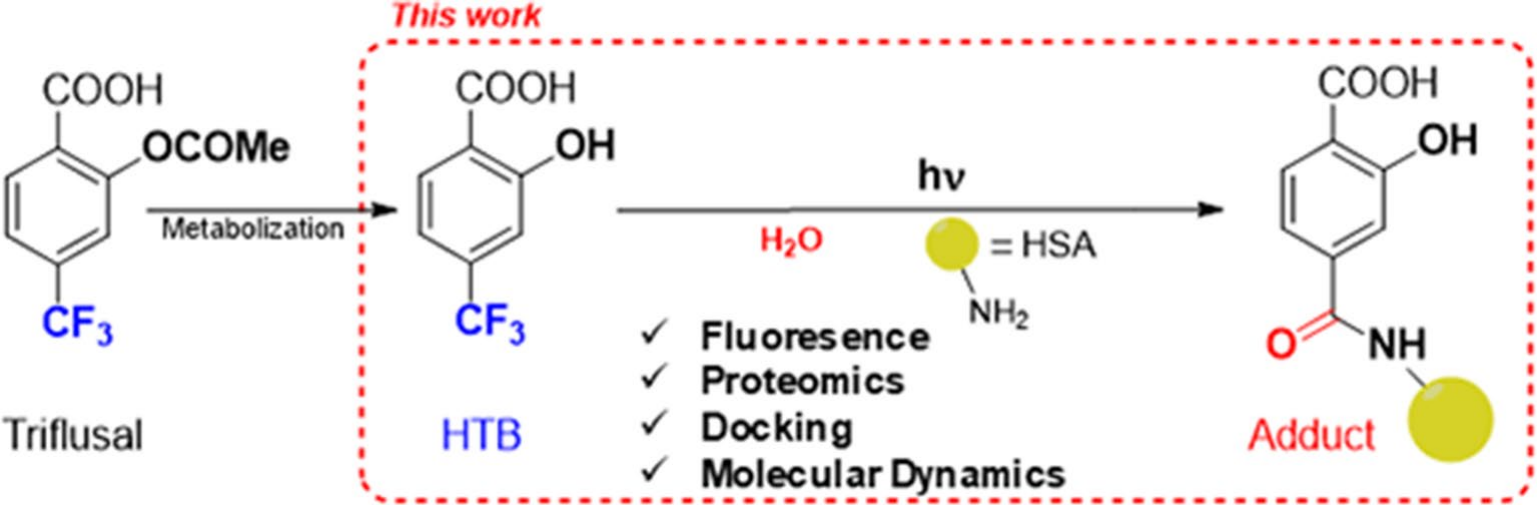
Approach for investigating the photobinding of HTB to HSA.

With this background, we decided to undertake an investigation on the possible modification of HSA lysine residues by photobinding of HTB. This will provide valuable information both on the molecular recognition center of the protein, and on the issue of HTB-mediated photoallergy. For this purpose, we have employed a combined fluorescence, proteomic and computational approach, as summarized in **Figure 1**.

## Results and Discussion

The obtained results are presented below, arranged in three sections dealing with size exclusion chromatography coupled with fluorescence measurements, proteomic analysis, and computational studies.

**Figure 2:**
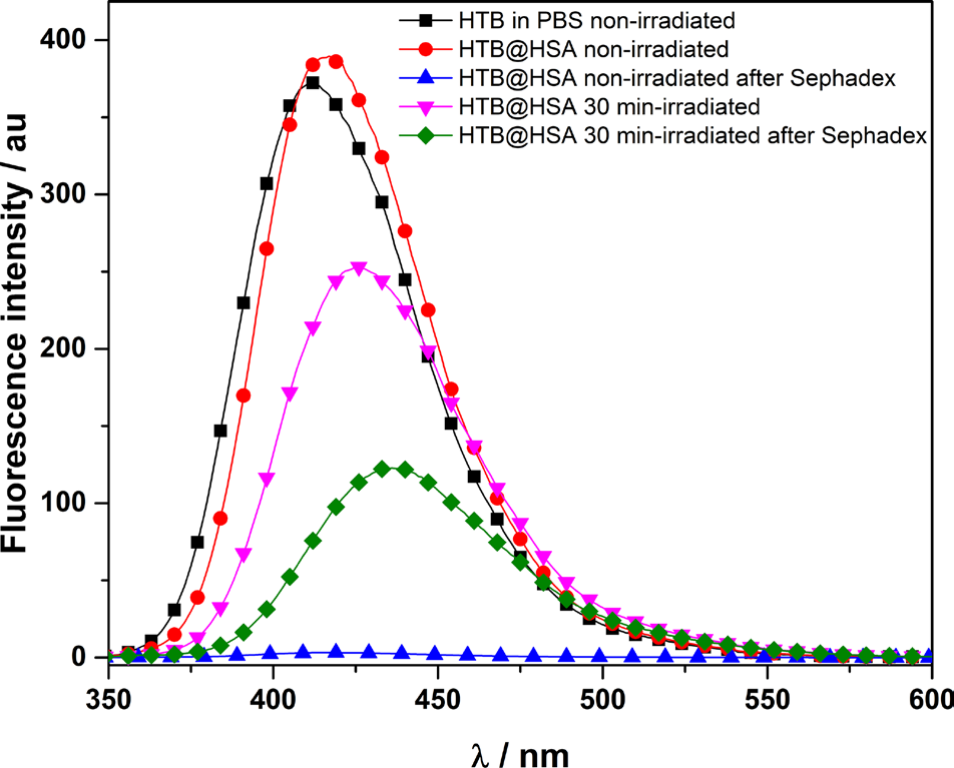
Fluorescence spectra (λ_exc_ = 320 nm) of non- and 30 min-irradiated samples of HTB alone and HTB/HSA mixtures, before and after treatment with guanidinium hydrochloride and Sephadex filtration. [HTB] = [HSA] = 5 × 10^−5^ M; PBS, air.

*Fluorescence detection of covalent HTB photoadducts to HSA*. A mixture of HTB (5 × 10^−5^ M) in the presence of HSA (1:1 drug/protein molar ratio) was irradiated in a multilamp photoreactor (λ_max_ = 300 nm, PBS, air, 30 min). The fluorescence spectra recorded before and after irradiation were markedly different (**Figure 2**). The band for the irradiated mixture was less intense and red-shifted (λ_max_ = 425 nm) respect to that non-irradiated (λ_max_ = 415 nm). This suggests photodegradation, in agreement with the previously reported photoreactivity for other trifluoromethyl-substituted substrates (Pérez-Ruiz et al., 2018). The irradiated sample was then treated with guanidinium hydrochloride and filtered through Sephadex, in order to elute only the high-molecular weight components of the photomixture. The emission of the eluate still displayed fluorescence (λ_max_ = 435 nm), clearly indicating covalent HTB photobinding to the protein. The fluorescence of a non-irradiated HTB/HSA mixtures filtered through Sephadex was negligible, indicating no photobinding.

*Determination of the modified amino acid residues by proteomic analysis*. The photobinding of HTB to HSA was then investigated by proteomic analysis, a tool that allows identifying which amino acids are modified when covalent binding to proteins occurs. For that purpose, an irradiated HTB/HSA mixture ([HTB] = [HSA] = 5 × 10^−5^ M, λ_max_ = 300 nm, PBS, air, 30 min) was filtered to remove HTB excess, submitted to trypsin digestion (to cleave peptide chains mainly at the carboxyl side of Lys or Arg residues, unless there is a neighboring Pro residue), and the resulting mixture was analyzed by HPLC-MS/MS, in order to investigate the modified peptide sequence and to undertake a detailed characterization of the HTB-HSA adducts. Processing of the full scan and fragmentation data files was performed by using the Mascot^®^ database search engine, and by entering variable modifications that take into account Lys as the main nucleophilic sites able to react with the trifluoromethyl group of HTB.

**Figure 3:**
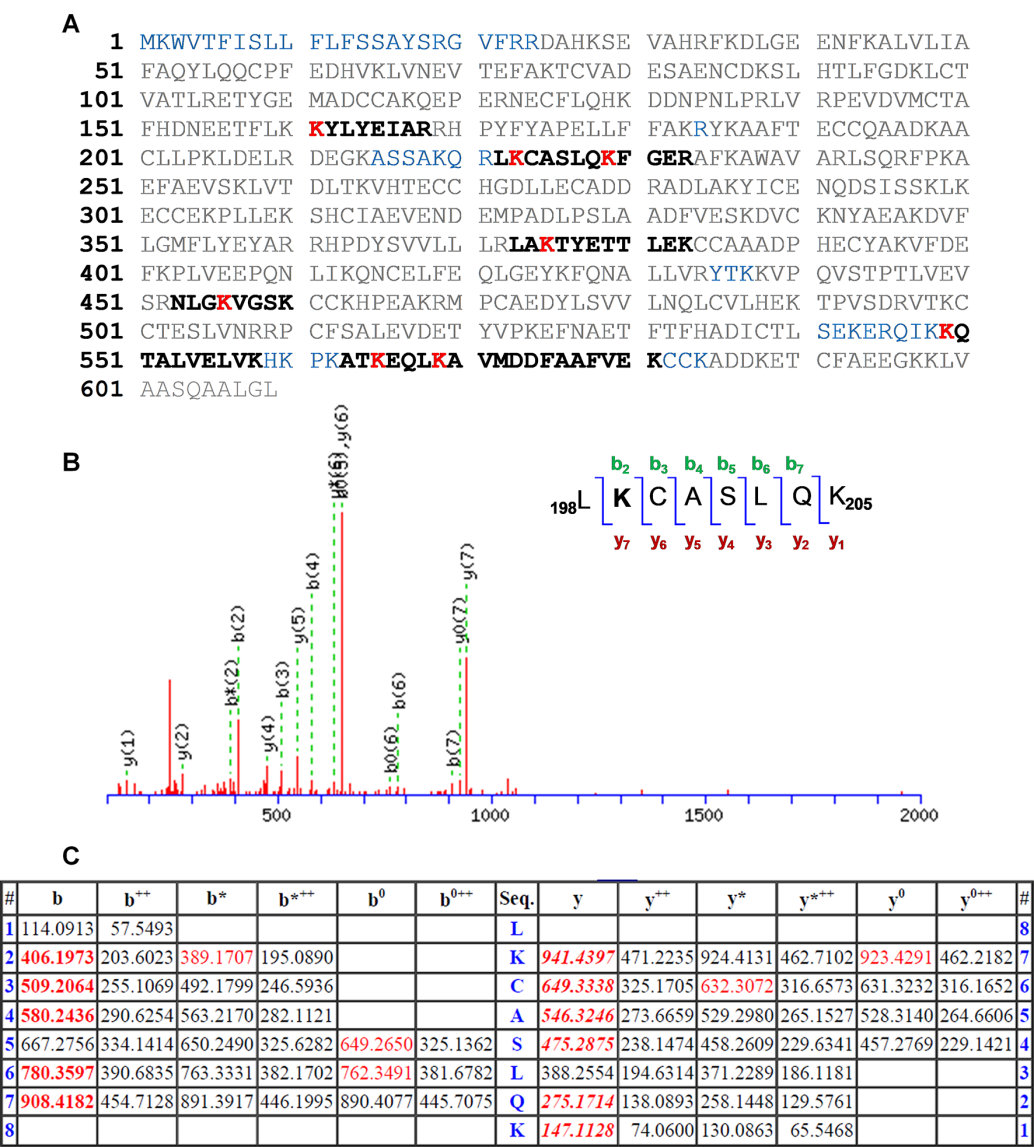
**(A)** Amino acid sequence (92% coverage) obtained after irradiation of HTB in the presence of HSA, with the non-resolved amino acids in blue. UniProtKB access number for HSA is P02768. The modified peptides are in black, and the altered amino acid residues are in red. **(B)** Modified peptide with fragmentation ions and ESI-MS/MS assigned spectrum of _198_L**K**CASLQK_205_. **(C)** Related data with the “y” and “b” ion series. Nomenclature for the different ion types is described in http://www.matrixscience.com/help/fragmentation_help.html.

The results are depicted in **Figure 3A** with the modified peptides in black and the modified amino acids in red. For clarity, the amino acid numbering used corresponds to that provided in PDB structures, where the first 24 amino acids are usually not observed. An increment of *ca.* 164 amu was observed in eight peptides. Formation of HTB-HSA adducts at Lys137, Lys199, Lys205, Lys351, Lys432, Lys525, Lys541, and Lys545 (**Table 1**) agrees with the ESI-MS/MS assigned spectra and fragmentation pattern of the modified peptides (see **Figures 3B, C** for Lys199 and **Figures S1–S7** in the ESI for the other modified Lys). The original ESI-HRMS/MS spectra of the modified peptides and tables containing the list of ions detected in the ESI-HRMS/MS spectra can be found in the ESI (**Figures S8-S15** and **Tables S1-S8**).

**Table 1:** Modified peptides, with experimental and calculated mass values.

Peptide	Mr exp	Mr calc	Modified Lys
_137_KYLYEIAR_144_	1,218.5930	1,218.5920	137
_198_LKCASLQK_205_	1,053.5158	1,053.5164	199
_200_CASLQKFGER_209_	1,358.5912	1,358.5925	205
_349_LAKTYETTLEK_359_	1,459.7046	1,459.7082	351
_429_NLGKVGSK_436_	965.48400	965.48180	432
_525_KQTALVELVK_534_	1,291.7032	1,291.7023	525
_539_ATKEQLK_545_	980.47860	980.48140	541
_542_EQLKAVMDDFAAFVEK_557_	2,003.9174	2,003.9186	545

**Figure 4:**
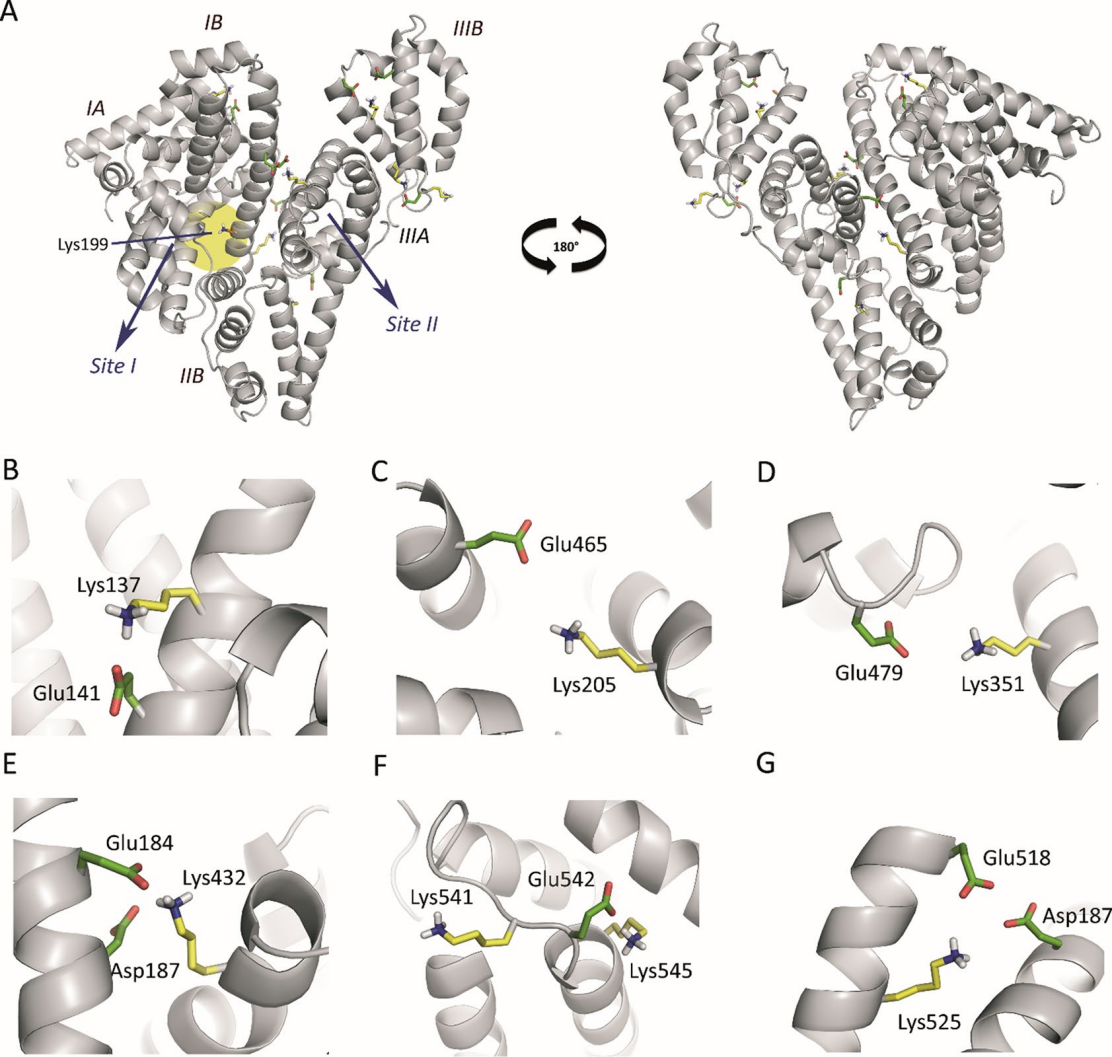
**(A)** Position of the Lys residues modified by HTB in the three-dimensional structure of HSA. Two perspectives are shown. The side chains of the external modified Lys residues (yellow), the internal Lys199 (orange), and the acidic residues constituting the local environment of the external Lys residues (Glu/Asp, green) are indicated. **(B**−**G)** Close view of the seven modified external Lys residues (Lys137, Lys205, Lys351, Lys541, Lys545, Lys525, and Lys432) identified by proteomic analysis. Note how, for all cases, the latter residues have either Glu or Asp residues in the vicinity to deprotonate them. The position of the internal lysine residue Lys199 is highlighted with a yellow shadow.

An analysis of the arrangement of the Lys residues covalently modified by HTB (Lys137, Lys199, Lys205, Lys351, Lys432, Lys525, Lys541 and Lys545) in the tridimensional structure of HSA, revealed that: i) the vast majority of Lys residues present in HSA (60) remain unmodified, ii) only Lys199 is located in an internal pocket of the protein, and iii) the remaining modified residues are placed in the external part of the protein (**Figure 4**). The covalent modification of these lysine residues was further studied at atomic detail by docking and molecular dynamics (MD) simulation studies, which is discussed below.

*Computational studies to elucidate the HTB binding mode*. It has been reported that Lys199 has an unusually low p*K*_a_ of ∼8 that favors a neutral protonation state (nucleophile), allowing its chemical modification by electrophilic reagents, such as trifluoromethyl-substituted aryl halides and sulfonates (Gerig and Reinheimer, 1975; Gerig et al., 1978). In addition, diverse experimental results and computational studies have identified Lys199 as the key catalytic residue in the esterase activity of HSA (**Figure 4A**) (Gerig et al., 1981; Díaz et al., 2001; Lockridge et al., 2008; Phuangsawai et al., 2014). Interestingly, all the external Lys residues modified by HTB have an acidic residue(s) (Glu/Asp) in their local environments that can act as general base for deprotonation and therefore the generation of the nucleophile, which justify the experimentally observed covalent modifications of these external lysine residues, corresponding to non-specific binding of HTB to HSA (**Figures 4B−G**).

The selective covalent modification of Lys199 by HTB among all the internal lysine residues in HSA could be due to binding of the ligand in the identified pocket of the protein; in an effort to understand in atomic detail the HTB binding mode, computational studies were performed. To this end, molecular docking using GOLD 5.2.2 (Jones et al., 1997) was carried out using the available protein coordinates of the crystallographically determined HSA in complex with oxyphenbutazone (PDB code 2BXB) (Ghuman et al., 2005). This structure was selected considering the results of our previous studies with quinone methides generated by photoirradiation (Pérez-Ruiz et al., 2018). These reactive intermediates proved to cause the covalent modification of Lys195 that is located in the vicinity of Lys199. The proposed binding mode of HTB was further validated by MD simulation studies that were performed by using the monomer of the HTB@HSA protein complex obtained by docking in a truncated octahedron of water molecules obtained with the molecular mechanics force field Amber (Case et al., 2005).

**Figure 5:**
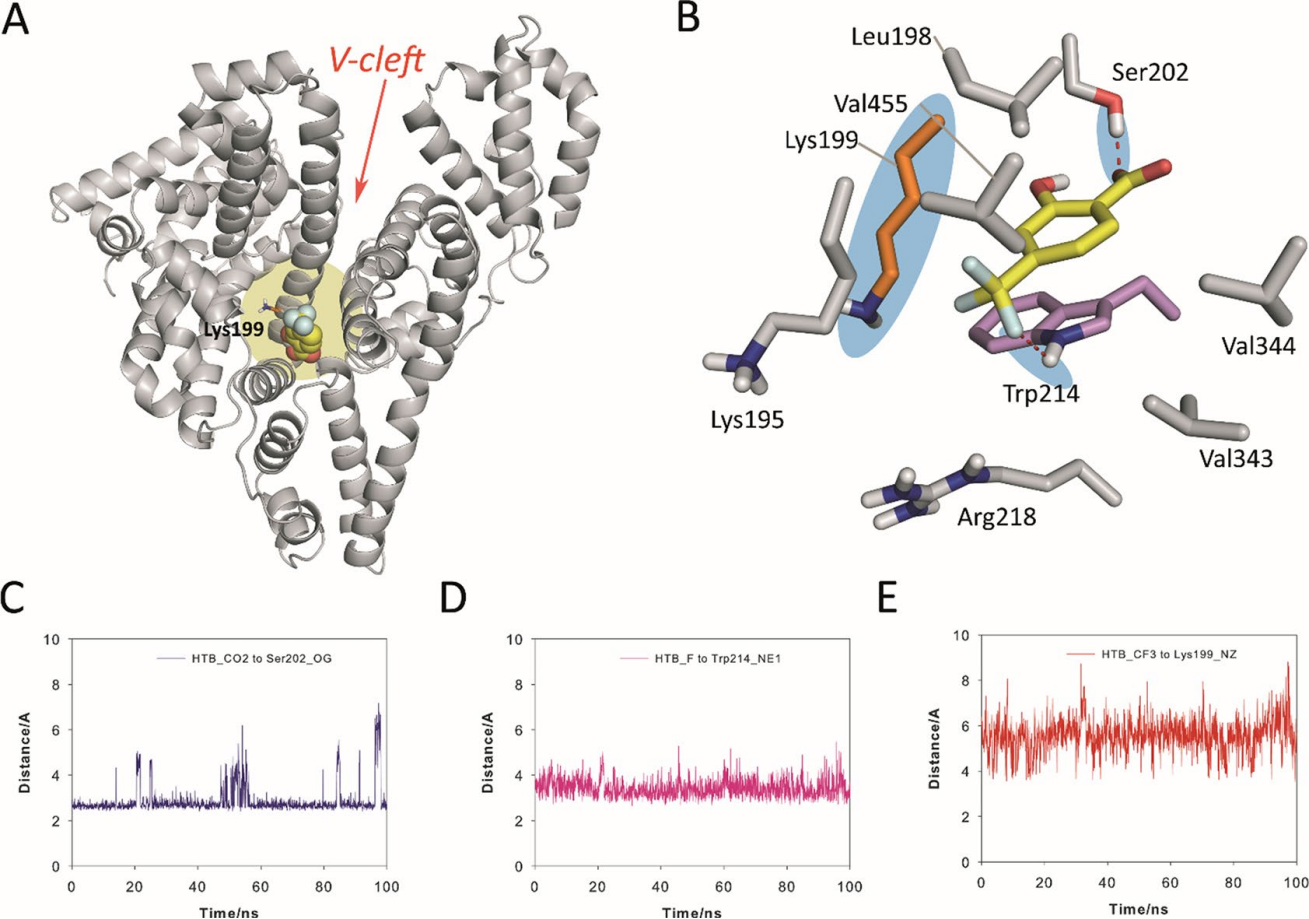
Proposed binding mode of HTB (yellow) to HSA protein as obtained by MD simulation studies. **(A)** Overall view of the proposed binary HTB@HSA complex. Snapshot after 88 ns is shown. The side chain of the experimentally modified internal lysine residue is shown (orange). **(B)** Detailed view of the HTB@HSA complex. The position of Lys199 and relevant hydrogen bonds are highlighted with a blue shadow. **(C, D, E)** Variation of the relative distance in the HTB@HSA protein complex during 100 ns of simulation between: **(C)** the O12 atom (CO_2_ group) in HTB and the side-chain oxygen atom (OG) of Ser202, **(D)** the closest fluorine atom (CF_3_ group) in HTB and the aromatic nitrogen atom (NE1) of Trp214, and **(E)** the C13 atom (CF_3_ group) in HTB and the NZ atom of Lys199. Note how Lys199 is well located for nucleophilic attack to the CF_3_ group. Relevant side-chain residues are shown and labeled. Hydrogen-bonding interactions are shown as red dashed lines. The position of Lys199 and relevant hydrogen bonds are highlighted with a blue shadow.

The results from the simulation studies showed that the proposed binding for HSA in the “V-cleft” region obtained by docking was reliable as the HTB@HSA-V-cleft complex proved to be very stable during 100 ns of simulation (**Figure 5**). Thus, an analysis of the root-mean-square deviation (rmsd) for the protein backbone (Cα, C, N, and O atoms) calculated for the complex obtained from MD simulation studies (100 ns) revealed average values of 1.8 Å (see **Figure S16** in the ESI). In addition, no significant modifications in the position of the ligand were observed during the simulation (average 1.6 Å). HTB would be anchored to the “V-cleft” pocket of HSA by two strong hydrogen-bonding interactions, specifically, one between the carboxylate group in HTB and the side chain of Ser202 and the second one between a fluorine atoms of CF_3_ in HTB and the NH group of Trp214 (**Figures 5A, B**). The stability of these interactions can be easily visualized by an analysis of the relative distance between the atoms involved in those interactions as it is shown in **Figures 5C, D**. Thus, the average distances between the O12 atom (CO_2_ group) in HTB and the side-chain oxygen atom (OG) in Ser202, and the closest fluorine atom in HTB and the aromatic nitrogen atom (NE1) in Trp214 were 2.9 Å and 3.4 Å, respectively. In addition, the aromatic ring of the ligand would be embedded in the apolar pocket involving the side chains of residues Leu198, Val455, Val344, Val343, Trp214, and Lys195 (carbon chain). It is worth noting the strong π-stacking interaction between the ligand and the indole ring of Trp214, which are stacked at a distance of about 4 Å during the whole simulation.

Importantly, the results of our computational studies would also explain the covalent modification of Lys199. Thus, (i) the CF_3_ moiety in HTB would be located in the proximity of ε-amino group of Lys199 with an average distance (between N and C atoms) of 5.5 Å during the simulation (**Figure 5E**), and (ii) both the nucleophile and the CF_3_ group in HTB would have the appropriate arrangement for the nucleophilic substitution reaction that triggers the amide adduct formation (**Figure 5B**). Moreover, these results were also in agreement with the previously reported displacement studies using ibuprofen, which is a non-steroidal anti-inflammatory drug with high affinity to site II, pointing to site I as clearly preferred for binding of HTB (Montanaro et al., 2009).

## Conclusions

The active metabolite of triflusal, HTB, undergoes covalent photobinding to HSA. The amino acids that become modified in the process are eight Lys residues of the protein, seven of them are external, and only one (Lys 199) is located in a binding pocket of HSA. The mass spectrometric analysis of the adducts is consistent with photonucleophilic attack of the ε-amino group of Lys to the trifluoromethyl group of HTB, which is assisted by the presence of Glu/Asp residues in the neighborhood of the external Lys units. Based on docking and MD simulation studies, the HTB binding domain to HSA has been identified in atomic detail, and the covalent modification mechanism triggered upon irradiation can be explained. Thus, HTB is anchored to the “V-cleft” pocket of HSA by two strong hydrogen-bonding interactions with Ser202 and Trp214, with its aromatic ring embedded in the apolar pocket involving the Leu198, Val455, Val344, Val343, Trp214, and Lys195 residues. Overall, the obtained results explain the covalent modification of Lys199 and are relevant in connection with the photoallergy observed for triflusal in clinical studies.

## Experimental Section

**General**. 2-Hydroxy-4-trifluoromethylbenzoic acid (HTB) and HSA were commercially available from Merck. Spectrophotometric, HPLC, or reagent grade solvents were used without further purification. Solutions of phosphate-buffered saline (PBS) (0.01 M, pH 7.4) were prepared by dissolving phosphate-buffered saline tablets in Milli-Q water.

**Fluorescence Experiments**. Spectra were recorded on a JASCO FP-8500 spectrofluorometer system, provided with a monochromator in the wavelength range of 200−850 nm, at 22°C. Experiments were performed on solutions of HTB (5 × 10^−5^ M) in the presence of HSA (at 1:1 HTB/HSA molar ratio), employing 10 × 10 mm^2^ quartz cells with 4-ml capacity.

**Steady-State Photolysis Experiments**. Steady-state photolysis of HTB (5 × 10^−5^ M) was performed by using a 150 W Xe Lamp coupled to a monochromator at lamp output (l_exc_ = 300 nm) in PBS under air and in the presence of protein (HTB/HSA 1:1 molar ratio), through Pyrex. The course of the reaction was followed by monitoring the changes in the fluorescence spectra of the reaction mixtures at increasing times.

**Treatment with Guanidinium Chloride and Filtration through Sephadex**. Guanidinium chloride (1.72 ml, 6 M) was added to 3 ml of HTB/HSA in PBS, in order to cause protein denaturation. The mixture was then filtered through a PD-10 desalting column containing 8.3 ml of Sephadex™ G-25 medium. Firstly, 25 ml of pure PBS were eluted; then, 2.5 ml of the HTB/HSA mixture treated with GndCl were also eluted; finally, 3.5 ml of PBS were eluted again. The absorption and emission of the final sample were then measured. To take into account the dilution factor, a similar experiment was conducted directly on HSA (in the absence of HTB). In this way, the ratio between the absorbance value before and after filtration was obtained, which was employed as correction factor in the experiments.

**Protein Digestion and LC-ESI-MS/MS Analysis**. The proteic contents of irradiated samples were enzymatically digested into smaller peptides using trypsin. Subsequently, these peptides were analyzed using nanoscale liquid chromatography coupled to tandem mass spectrometry (NanoLC-MS/MS). Briefly, 20 µg of sample were taken (according to Qubit quantitation), and the volume was set to 20 µl. Digestion was achieved with sequencing grade trypsin (Promega) according to the following steps: i) 2 mM DTT in 50 mM NH_4_HCO_3_ V = 25 µl, 20 min (60°C); ii) alkylation of thiol groups by 5.5 mM IAM in 50 mM NH_4_HCO_3_ V = 30 µl, 30 min (dark); iii) 10 mM DTT in 50 mM NH_4_HCO_3_ V = 60 µl, 30 min; and iv) trypsin (trypsin: protein ratio 1:20 w/w) V = 64 µl, overnight 37°C. Digestion was stopped with 7 µl 10% TFA (Cf protein ca 0.28 µg/µl). Next, 5 µl of sample were loaded onto a trap column (NanoLC Column, 3µ C18-CL, 350 µm × 0.5 mm; Eksigent) and desalted with 0.1% TFA at 3 µl/min during 5 min. The peptides were then loaded onto an analytical column (LC Column, 3 µ C18-CL, 75 µm × 12 cm, Nikkyo) equilibrated in 5% acetonitrile 0.1% formic acid. Elution was carried out with a linear gradient of 5% to 45% B in A for 30 min (A: 0.1% formic acid; B: acetonitrile, 0.1% formic acid) at a flow rate of 300 µl/min. Peptides were analyzed in a mass spectrometer nanoESI-qQTOF (5600 TripleTOF, ABSCIEX). The TripleTOF was operated in information-dependent acquisition mode, in which a 0.25-s TOF MS scan from 350–1,250 m/z was performed, followed by 0.05-s product ion scans from 100–1,500 m/z on the 50 most intense 2–5 charged ions. ProteinPilot v4.5. (AB Sciex) search engine default parameters were used to generate peak list directly from 5600 TripleTOF wiff files. The obtained mgf was used for identification with MASCOT (v 4.0, Matrix Science). Database search was performed on Swiss-Prot database. Searches were done with tryptic specificity allowing one missed cleavage and a tolerance on the mass measurement of 100 ppm in MS mode and 0.6 Da in MS/MS mode. Carbamidomethylation of cysteine residues is defined as fixed modification. The modification due to this reagent was defined in the MASCOT server.

**Docking Studies**. They were carried out using program GOLD 5.2.2 and the protein coordinates found in the crystal structure of HSA in complex with oxyphenbutazone (PDB code 2BXB). Ligand geometries were minimized using the AM1 Hamiltonian as implemented in the program Gaussian 09 and used as MOL2 files. Each ligand was docked in 25 independent genetic algorithm (GA) runs, and for each of these, a maximum number of 100,000 GA operations were performed on a single population of 50 individuals. Operator weights for crossover, mutation, and migration in the entry box were used as default parameters (95, 95, and 10, respectively) as well as the hydrogen bonding (4.0 Å) and van der Waals (2.5 Å) parameters. The position of the side chain of the experimentally observed modified residue was used to define the active site, and the radius was set to 8 Å. All crystallographic water molecules and the ligands were removed for docking. The “flip ring corners” flag was switched on, while all the other flags were off. The GOLD scoring function was used to rank the ligands for fitting.

**Molecular Dynamics Simulations Studies**. *Ligand Minimization*. The ligand geometries of the highest score solution obtained by docking were minimized using a restricted Hartree−Fock (RHF) method and a 6-31G(d) basis set, as implemented in the *ab initio* program Gaussian 09 (Frisch et al., 2009). Partial charges were derived by quantum mechanical calculations using Gaussian 09, as implemented in the R.E.D. Server (version 3.0) (Dupradeau et al., 2010; Vanquelef et al., 2011), according to the RESP (Cornell et al., 1995) model. Ligand coordinates obtained by docking were employed as starting point for MD simulations. The missing bonded and nonbonded parameters were assigned, by analogy or through interpolation, from those already present in the Amber database (GAFF) (Wang et al., 2004; Wang et al., 2006).

*Generation and Minimization of the Complexes*. Simulations of the HTB@HSA binary complex were carried out using the enzyme geometries in PDB code 2BXB and the ligand geometries of the highest score solution. Computation of the protonation state of titratable groups at pH 7.0 was carried out using the H^++^ Web server (Gordon et al., 2005). Addition of hydrogen and molecular mechanics parameters from the ff14SB (Maier et al., 2015) and GAFF force fields, respectively, were assigned to the protein and the ligands using the LEAP module of AmberTools 17. As a result of this analysis: i) His535 was protonated in δ position; ii) His3, His9, His39, His146, His242, His288, His440, His464, and His510 were protonated in ε position; and iii) His67, His105, His128, His247, His338, and His367 were protonated in δ and ε positions. The protein was immersed in a truncated octahedron of ∼25,000 TIP3P water molecules and neutralized by addition of sodium ions. The system was minimized in five stages: a) minimization of poorly unsolved residues: 12, 33, 41, 51, 56, 60, 73, 81, 82, 84, 94, 95, 97, 111, 114, 174, 186, 190, 205, 209, 225, 227, 240, 250, 275, 276, 277, 297, 301, 313, 317, 321, 359, 390, 402, 436, 439, 444, 466, 513, 519, 524, 532, 536, 538, 541, 545, 550, 551, 560, 562, 564, 565, 574, and 580 (1,000 steps, first half using steepest descent and the rest using conjugate gradient); b) minimization of the ligand (1,000 steps, first half using steepest descent and the rest using conjugate gradient); c) minimization of the solvent and ions (5,000 steps, first half using steepest descent and the rest using conjugate gradient); d) minimization of the side-chain residues, waters, and ions (5,000 steps, first half using steepest descent and the rest using conjugate gradient); and e) final minimization of the whole system (5,000 steps, first half using steepest descent, and the rest using conjugate gradient). A positional restraint force of 50 kcal mol^−1^ Å^−2^ was applied to not minimized residues of the protein during the stages a−c and to α carbons during the stage d, respectively.

*Simulations*. MD simulations were performed using the pmemd.cuda_SPFP (Goetz et al., 2012; Le Grand et al., 2013; Salomon-Ferrer et al., 2013) module from the AMBER 16 suite of programs. Periodic boundary conditions were applied, and electrostatic interactions were treated using the smooth particle mesh Ewald method (PME) (Darden et al., 1993) with a grid spacing of 1 Å. The cutoff distance for the nonbonded interactions was 9 Å. The SHAKE algorithm (Ryckaert et al., 1977) was applied to all bonds containing hydrogen using a tolerance of 10^−5^ Å and an integration step of 2.0 fs. The minimized system was then heated at 300 K at 1 atm by increasing the temperature from 0 to 300 K over 100 ps and by keeping the system at 300 K another 100 ps. A positional restraint force of 50 kcal mol^−1^ Å^−2^ was applied to all α carbons during the heating stage. Finally, an equilibration of the system at constant volume (200 ps with positional restraints of 5 kcal mol^−1^ Å^−2^ to α alpha carbons) and constant pressure (another 100 ps with positional restraints of 5 kcal mol^−1^ Å^−2^ to α carbons) was performed. The positional restraints were gradually reduced from 5 to 1 mol^−1^ Å^−2^ (5 steps, 100 ps each), and the resulting systems were allowed to equilibrate further (100 ps). Unrestrained MD simulations were carried out for 100 ns. System coordinates were collected every 10 ps for further analysis. The molecular graphics program PyMOL (DeLano, 2008) was employed for visualization and depicting ligand/protein structures. The cpptraj module in AMBER 16 was used to analyze the trajectories and to calculate the rmsd of the protein during the simulation (Roe and Cheatham, 2013).

## Data Availability

The raw data supporting the conclusions of this manuscript will be made available by the authors, without undue reservation, to any qualified researcher.

## Author Contributions

All authors have realized substantial, direct, and intellectual contribution to the work, and approved it for publication.

## Conflict of Interest Statement

The authors declare that the research was conducted in the absence of any commercial or financial relationships that could be construed as a potential conflict of interest.
