# Supplementary material for: Photobinding of Triflusal to Human Serum Albumin Investigated by Fluorescence, Proteomic Analysis, and Computational Studies
Source: Front Pharmacol. 2019 Sep 20;10:1028. doi: 10.3389/fphar.2019.01028 (PMC6764118; doi:10.3389/fphar.2019.01028)
Supplement: Supplementary file 1 [file DataSheet_1.pdf]

# Photobinding of Triflusal to Human Serum Albumin Investigated by Fluorescence, Proteomic Analysis and Computational Studies.

Oscar Molins-Molina,[a] Raúl Pérez-Ruiz,[a] Emilio Lence,[b] Concepción González-Bello,[b] Miguel A. Miranda,*[a] and M. Consuelo Jiménez*[a]

[a] Dpto. de Química/Instituto de Tecnología Química UPV-CSIC Universitat Politécnica de València, Camino de Vera s/n 46071 Valencia (Spain).
[b] Centro Singular de Investigación en Química Biolóxica e Materiais Moleculares (CIQUS), Departamento de Química Orgánica, Universidade de Santiago de Compostela, c/ Jenaro de la Fuente s/n, 15782 Santiago de Compostela (Spain).

## Table of contents

-Page S1. This Page.

-Page S2. **Scheme S1.** A plausible mechanism for the photochemical reaction between Lys and HTB.

-Page S3. **Figure S1**. Modified peptide and ESI-MS/MS assigned spectrum of $_{137}$KYLYEIAR$_{144}$.

-Page S4. **Figure S2**. Modified peptide and ESI-MS/MS assigned spectrum of $_{200}$CASLQKFGER$_{209}$.

-Page S5. **Figure S3**. Modified peptide and ESI-MS/MS assigned spectrum of $_{349}$LAKTYETTLEK$_{359}$.

-Page S6. **Figure S4**. Modified peptide and ESI-MS/MS assigned spectrum of $_{429}$NLGKVGSR$_{436}$.

-Page S7. **Figure S5**. Modified peptide and ESI-MS/MS assigned spectrum of $_{525}$KQTALVELVK$_{534}$.

-Page S8. **Figure S6**. Modified peptide and ESI-MS/MS assigned spectrum of $_{539}$ATKEQLK$_{545}$.

-Page S9. **Figure S7**. Modified peptide and ESI-MS/MS assigned spectrum of $_{542}$EQLKAVMDDFAAFVEK$_{557}$.

-Pages S10-S17. **Figures S8-S15**. Original ESI-HRMS/MS spectra of the corresponding modified peptides.

-Pages S18-S27. **Tables S1-S8**. Tables containing the list of ions detected in the ESI-HRMS/MS spectra for the corresponding modified peptides.

-Page S28. **Figure S16**. The rmsd plots for the protein backbone (Cα, C, N and O atoms) (up) and ligand (down) calculated for the binary HTB@HSA complex obtained from MD simulation studies.

**Scheme S1.** A plausible mechanism for the photochemical reaction between Lys and HTB.

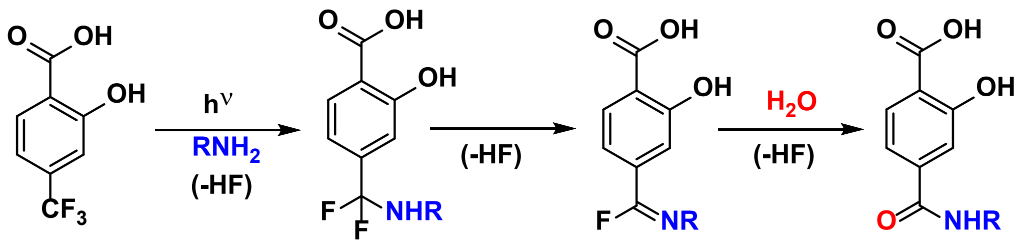

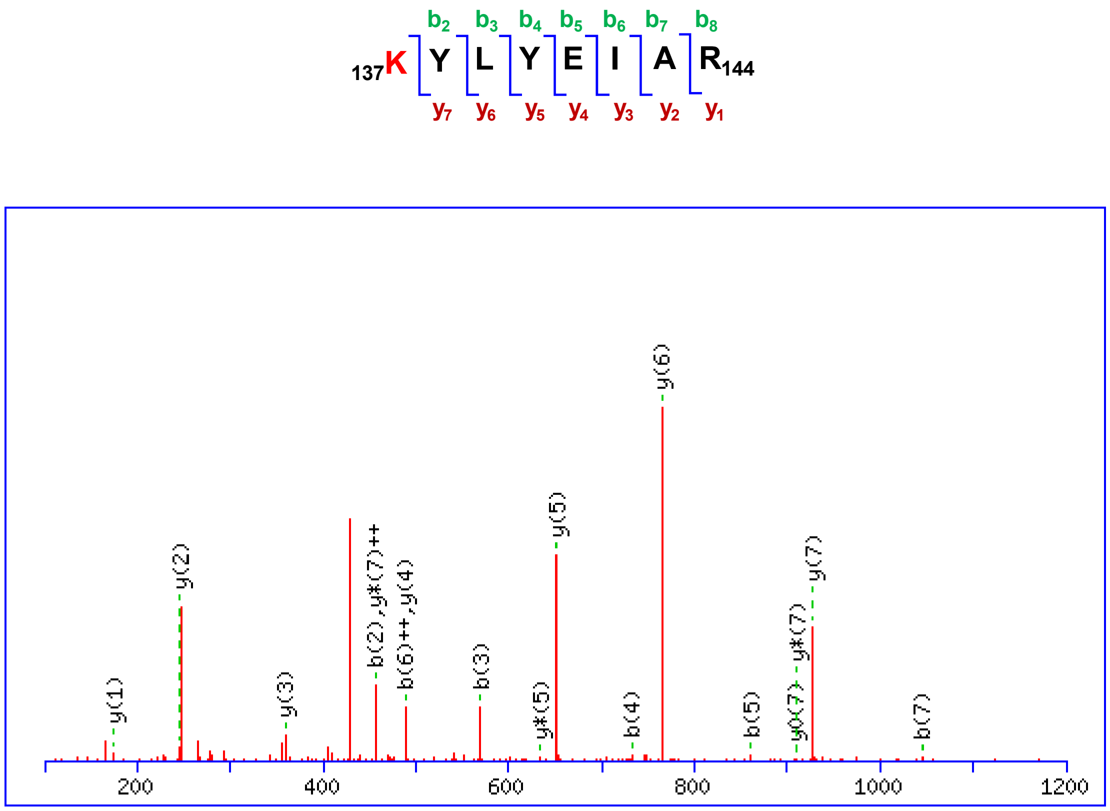


| # | b | $b^{++}$ | $b^{*}$ | $b^{*++}$ | $b^{0}$ | $b^{0++}$ | Seq. | y | $y^{++}$ | $y^{*}$ | $y^{*++}$ | $y^{0}$ | $y^{0++}$ | # |
|---|---|---|---|---|---|---|---|---|---|---|---|---|---|---|
| 1 | 293.1132 | 147.0602 | 276.0866 | 138.5470 | | | K | | | | | | | 8 |
| 2 | **456.1765** | 228.5919 | 439.1500 | 220.0786 | | | Y | ***927.4934*** | 464.2504 | 910.4669 | 455.7371 | 909.4829 | 455.2451 | 7 |
| 3 | **569.2606** | 285.1339 | 552.2340 | 276.6207 | | | L | ***764.4301*** | 382.7187 | 747.4036 | 374.2054 | 746.4196 | 373.7134 | 6 |
| 4 | **732.3239** | 366.6656 | 715.2974 | 358.1523 | | | Y | ***651.3461*** | 326.1767 | 634.3195 | 317.6634 | 633.3355 | 317.1714 | 5 |
| 5 | **861.3665** | 431.1869 | 844.3400 | 422.6736 | 843.3559 | 422.1816 | E | ***488.2827*** | 244.6450 | 471.2562 | 236.1317 | 470.2722 | 235.6397 | 4 |
| 6 | 974.4506 | 487.7289 | 957.4240 | 479.2157 | 956.4400 | 478.7236 | I | ***359.2401*** | 180.1237 | 342.2136 | 171.6104 | | | 3 |
| 7 | **1045.4877** | 523.2475 | 1028.4611 | 514.7342 | 1027.4771 | 514.2422 | A | ***246.1561*** | 123.5817 | 229.1295 | 115.0684 | | | 2 |
| 8 | | | | | | | R | ***175.1190*** | 88.0631 | 158.0924 | 79.5498 | | | 1 |

**Figure S1.** Modified peptide and ESI-MS/MS assigned spectrum of $_{137}$KYLYEIAR$_{144}$. For clarity, the amino acid numbering used corresponds to the provided in PDB structures as the first 24 amino acids are usually not observed.

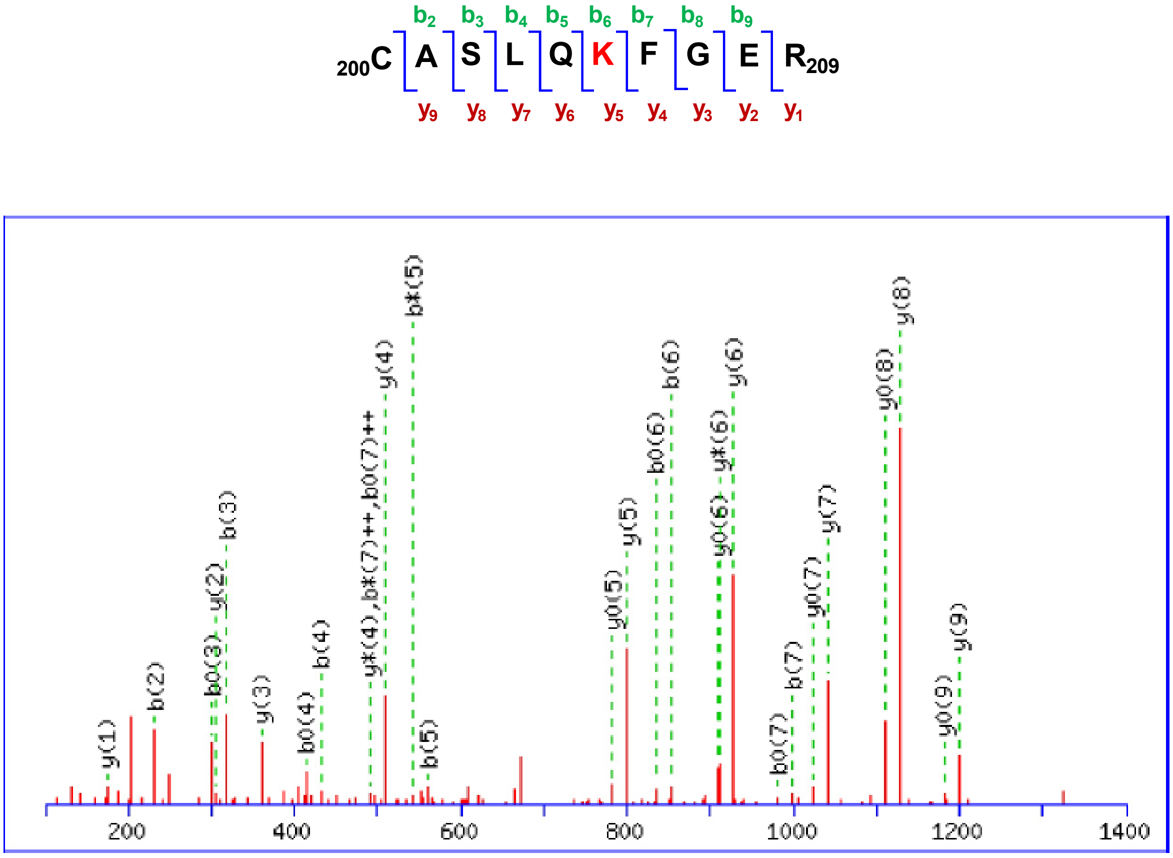


| # | b | $b^{++}$ | $b^{*}$ | $b^{*++}$ | $b^{0}$ | $b^{0++}$ | Seq. | y | $y^{++}$ | $y^{*}$ | $y^{*++}$ | $y^{0}$ | $y^{0++}$ | # |
|---|---|---|---|---|---|---|---|---|---|---|---|---|---|---|
| 1 | 161.0379 | 81.0226 | | | | | C | | | | | | | 10 |
| 2 | **232.0750** | 116.5412 | | | | | A | ***1199.5691*** | 600.2882 | 1182.5426 | 591.7749 | 1181.5586 | 591.2829 | 9 |
| 3 | **319.1071** | 160.0572 | | | 301.0965 | 151.0519 | S | ***1128.5320*** | 564.7696 | 1111.5055 | 556.2564 | 1110.5215 | 555.7644 | 8 |
| 4 | **432.1911** | 216.5992 | | | 414.1806 | 207.5939 | L | ***1041.5000*** | 521.2536 | 1024.4734 | 512.7404 | 1023.4894 | 512.2483 | 7 |
| 5 | **560.2497** | 280.6285 | 543.2232 | 272.1152 | 542.2391 | 271.6232 | Q | ***928.4159*** | 464.7116 | 911.3894 | 456.1983 | 910.4054 | 455.7063 | 6 |
| 6 | **852.3556** | 426.6815 | 835.3291 | 418.1682 | 834.3451 | 417.6762 | K | ***800.3573*** | 400.6823 | 783.3308 | 392.1690 | 782.3468 | 391.6770 | 5 |
| 7 | **999.4240** | 500.2157 | 982.3975 | 491.7024 | 981.4135 | 491.2104 | F | ***508.2514*** | 254.6293 | 491.2249 | 246.1161 | 490.2409 | 245.6241 | 4 |
| 8 | 1056.4455 | 528.7264 | 1039.4190 | 520.2131 | 1038.4349 | 519.7211 | G | ***361.1830*** | 181.0951 | 344.1565 | 172.5819 | 343.1724 | 172.0899 | 3 |
| 9 | 1185.4881 | 593.2477 | 1168.4616 | 584.7344 | 1167.4775 | 584.2424 | E | ***304.1615*** | 152.5844 | 287.1350 | 144.0711 | 286.1510 | 143.5791 | 2 |
| 10 | | | | | | | R | ***175.1190*** | 88.0631 | 158.0924 | 79.5498 | | | 1 |

**Figure S2.** Modified peptide and ESI-MS/MS assigned spectrum of $_{200}$CASLQKFGER$_{209}$. For clarity, the amino acid numbering used corresponds to the provided in PDB structures as the first 24 amino acids are usually not observed.

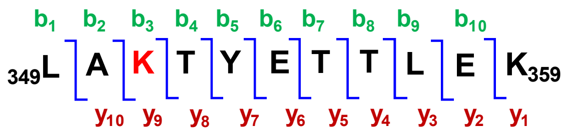


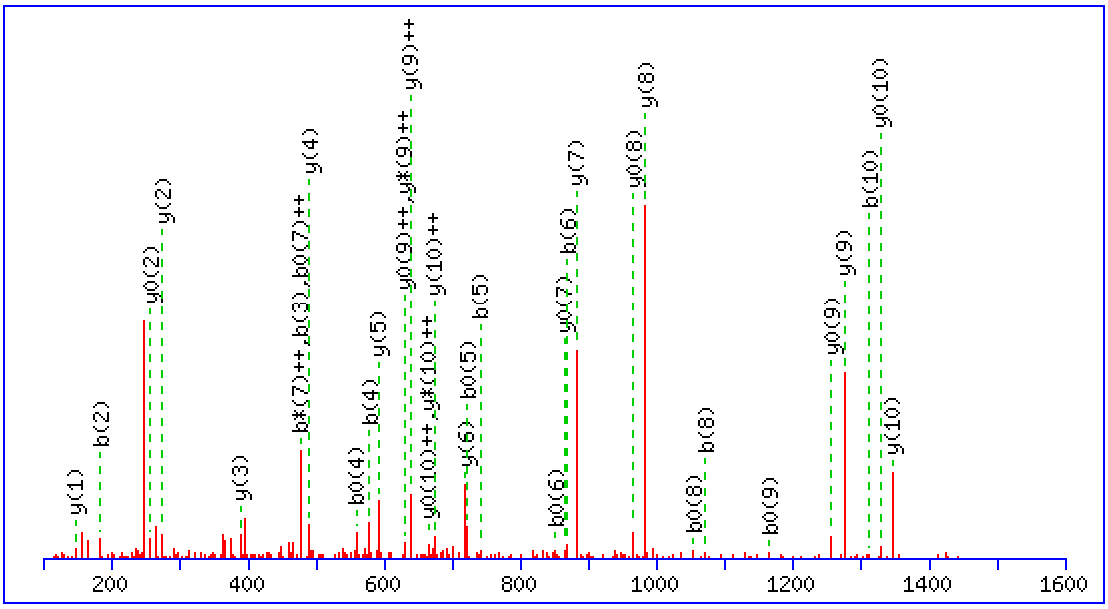

| # | b | $b^{++}$ | b* | $b^{*++}$ | $b^0$ | $b^{0++}$ | Seq. | y | $y^{++}$ | y* | $y^{*++}$ | $y^0$ | $y^{0++}$ | # |
|---|---|---|---|---|---|---|---|---|---|---|---|---|---|---|
| 1 | 114.0913 | 57.5493 | | | | | L | | | | | | | 11 |
| 2 | **185.1285** | 93.0679 | | | | | A | ***1347.6315*** | 674.3194 | 1330.6049 | 665.8061 | 1329.6209 | 665.3141 | 10 |
| 3 | **477.2344** | 239.1208 | 460.2078 | 230.6076 | | | K | ***1276.5943*** | 638.8008 | 1259.5678 | 630.2875 | 1258.5838 | 629.7955 | 9 |
| 4 | **578.2821** | 289.6447 | 561.2555 | 281.1314 | 560.2715 | 280.6394 | T | ***984.4884*** | 492.7478 | 967.4619 | 484.2346 | 966.4779 | 483.7426 | 8 |
| 5 | **741.3454** | 371.1763 | 724.3188 | 362.6631 | 723.3348 | 362.1710 | Y | ***883.4407*** | 442.2240 | 866.4142 | 433.7107 | 865.4302 | 433.2187 | 7 |
| 6 | **870.3880** | 435.6976 | 853.3614 | 427.1844 | 852.3774 | 426.6923 | E | ***720.3774*** | 360.6923 | 703.3509 | 352.1791 | 702.3668 | 351.6871 | 6 |
| 7 | 971.4357 | 486.2215 | 954.4091 | 477.7082 | 953.4251 | 477.2162 | T | ***591.3348*** | 296.1710 | 574.3083 | 287.6578 | 573.3243 | 287.1658 | 5 |
| 8 | **1072.4833** | 536.7453 | 1055.4568 | 528.2320 | 1054.4728 | 527.7400 | T | ***490.2871*** | 245.6472 | 473.2606 | 237.1339 | 472.2766 | 236.6419 | 4 |
| 9 | 1185.5674 | 593.2873 | 1168.5408 | 584.7741 | 1167.5568 | 584.2821 | L | ***389.2395*** | 195.1234 | 372.2129 | 186.6101 | 371.2289 | 186.1181 | 3 |
| 10 | **1314.6100** | 657.8086 | 1297.5834 | 649.2954 | 1296.5994 | 648.8034 | E | ***276.1554*** | 138.5813 | 259.1288 | 130.0681 | 258.1448 | 129.5761 | 2 |
| 11 | | | | | | | K | ***147.1128*** | 74.0600 | 130.0863 | 65.5468 | | | 1 |

**Figure S3.** Modified peptide and ESI-MS/MS assigned spectrum of ${}_{349}$LAKTYETTLEK${}_{359}$. For clarity, the amino acid numbering used corresponds to the provided in PDB structures as the first 24 amino acids are usually not observed.

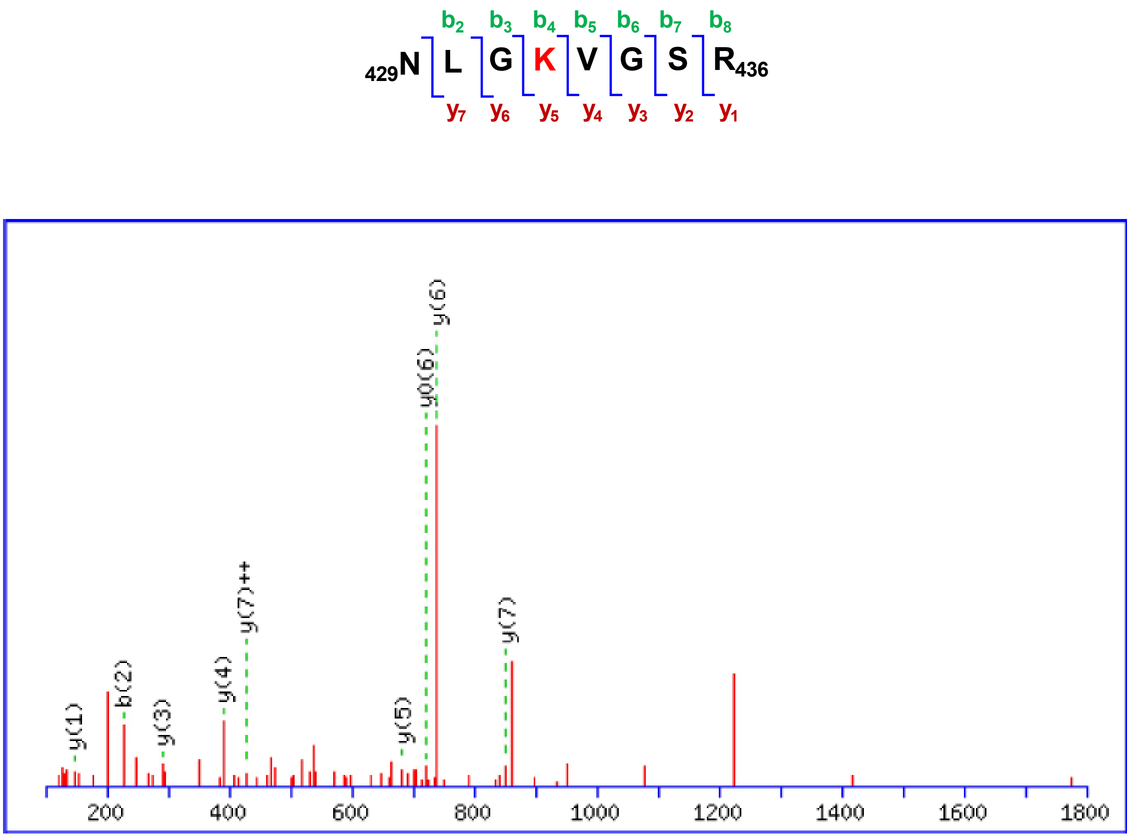


| # | b | b++ | b* | b*++ | b0 | b0++ | Seq. | y | y++ | y* | y*++ | y0 | y0++ | # |
|---|---|---|---|---|---|---|---|---|---|---|---|---|---|---|
| 1 | 115.0502 | 58.0287 | 98.0237 | 49.5155 | | | N | | | | | | | 8 |
| 2 | 228.1343 | 114.5708 | 211.1077 | 106.0575 | | | L | *852.4462* | 426.7267 | 835.4196 | 418.2134 | 834.4356 | 417.7214 | 7 |
| 3 | 285.1557 | 143.0815 | 268.1292 | 134.5682 | | | G | *739.3621* | 370.1847 | 722.3355 | 361.6714 | 721.3515 | 361.1794 | 6 |
| 4 | 577.2617 | 289.1345 | 560.2351 | 280.6212 | | | K | *682.3406* | 341.6740 | 665.3141 | 333.1607 | 664.3301 | 332.6687 | 5 |
| 5 | 676.3301 | 338.6687 | 659.3035 | 330.1554 | | | V | *390.2347* | 195.6210 | 373.2082 | 187.1077 | 372.2241 | 186.6157 | 4 |
| 6 | 733.3515 | 367.1794 | 716.3250 | 358.6661 | | | G | *291.1663* | 146.0868 | 274.1397 | 137.5735 | 273.1557 | 137.0815 | 3 |
| 7 | 820.3836 | 410.6954 | 803.3570 | 402.1821 | 802.3730 | 401.6901 | S | 234.1448 | 117.5761 | 217.1183 | 109.0628 | 216.1343 | 108.5708 | 2 |
| 8 | | | | | | | K | *147.1128* | 74.0600 | 130.0863 | 65.5468 | | | 1 |

**Figure S4.** Modified peptide and ESI-MS/MS assigned spectrum of $_{429}$NLGKVGSR$_{436}$. For clarity, the amino acid numbering used corresponds to the provided in PDB structures as the first 24 amino acids are usually not observed.

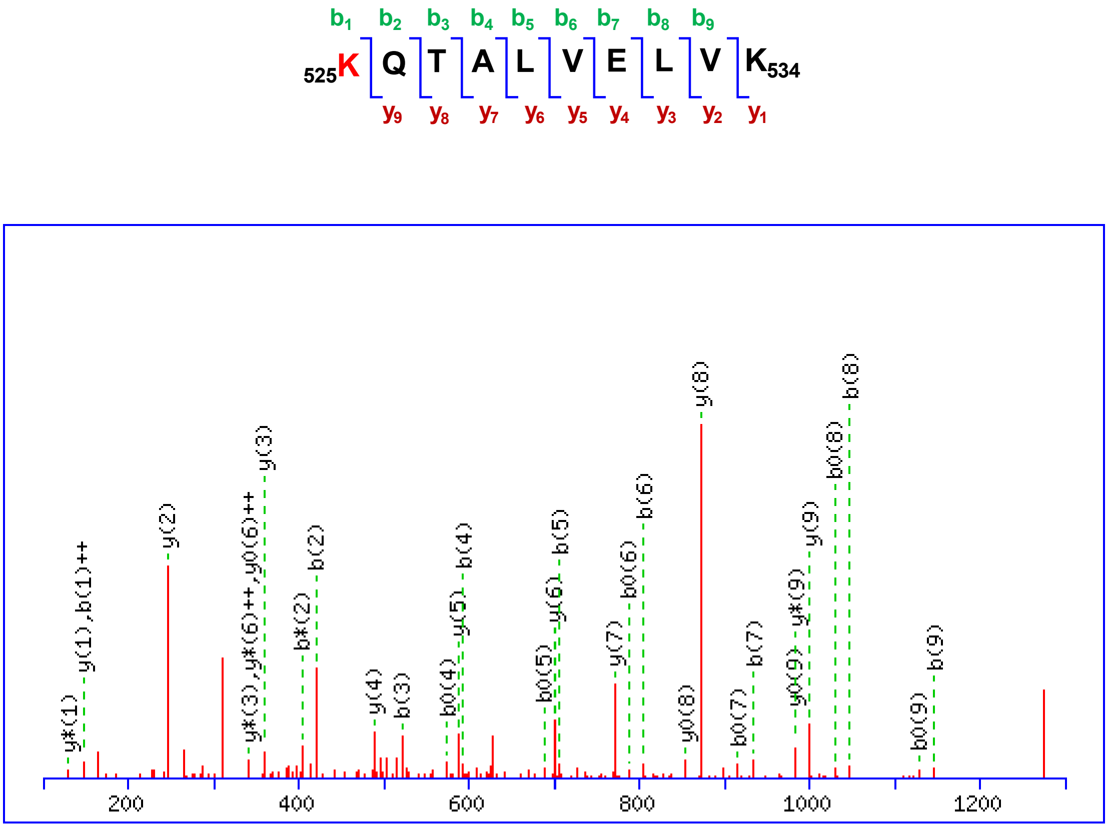


| # | b | $b^{++}$ | b* | $b^{*++}$ | $b^{0}$ | $b^{0++}$ | Seq. | y | $y^{++}$ | y* | $y^{*++}$ | $y^{0}$ | $y^{0++}$ | # |
|---|---|---|---|---|---|---|---|---|---|---|---|---|---|---|
| 1 | 293.1132 | 147.0602 | 276.0866 | 138.5470 | | | K | | | | | | | 10 |
| 2 | **421.1718** | 211.0895 | 404.1452 | 202.5763 | | | Q | ***1000.6037*** | 500.8055 | 983.5772 | 492.2922 | 982.5932 | 491.8002 | 9 |
| 3 | **522.2195** | 261.6134 | 505.1929 | 253.1001 | 504.2089 | 252.6081 | T | ***872.5451*** | 436.7762 | 855.5186 | 428.2629 | 854.5346 | 427.7709 | 8 |
| 4 | **593.2566** | 297.1319 | 576.2300 | 288.6186 | 575.2460 | 288.1266 | A | ***771.4975*** | 386.2524 | 754.4709 | 377.7391 | 753.4869 | 377.2471 | 7 |
| 5 | **706.3406** | 353.6740 | 689.3141 | 345.1607 | 688.3301 | 344.6687 | L | ***700.4604*** | 350.7338 | 683.4338 | 342.2205 | 682.4498 | 341.7285 | 6 |
| 6 | **805.4090** | 403.2082 | 788.3825 | 394.6949 | 787.3985 | 394.2029 | V | ***587.3763*** | 294.1918 | 570.3497 | 285.6785 | 569.3657 | 285.1865 | 5 |
| 7 | **934.4516** | 467.7295 | 917.4251 | 459.2162 | 916.4411 | 458.7242 | E | ***488.3079*** | 244.6576 | 471.2813 | 236.1443 | 470.2973 | 235.6523 | 4 |
| 8 | **1047.5357** | 524.2715 | 1030.5092 | 515.7582 | 1029.5251 | 515.2662 | L | ***359.2653*** | 180.1363 | 342.2387 | 171.6230 | | | 3 |
| 9 | **1146.6041** | 573.8057 | 1129.5776 | 565.2924 | 1128.5936 | 564.8004 | V | ***246.1812*** | 123.5942 | 229.1547 | 115.0810 | | | 2 |
| 10 | | | | | | | K | ***147.1128*** | 74.0600 | 130.0863 | 65.5468 | | | 1 |

**Figure S5.** Modified peptide and ESI-MS/MS assigned spectrum of $_{525}$KQTALVELVK$_{534}$. For clarity, the amino acid numbering used corresponds to the provided in PDB structures as the first 24 amino acids are usually not observed.

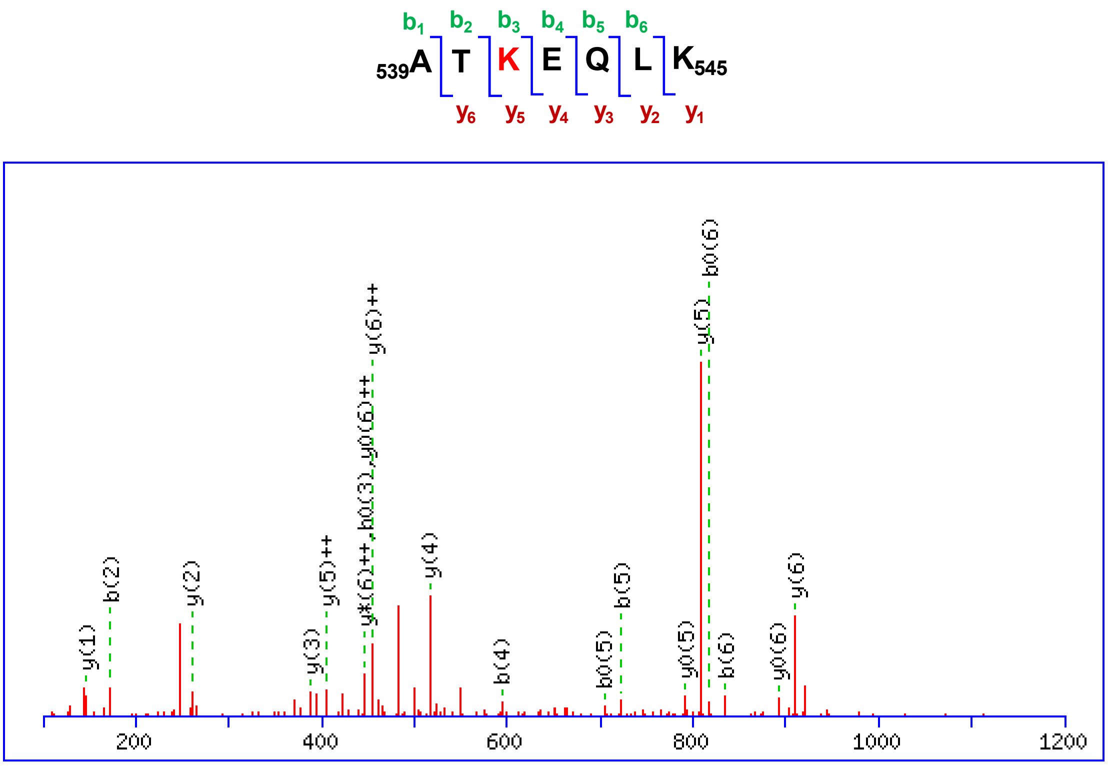


| # | b | $b^{++}$ | b* | $b^{*++}$ | $b^{0}$ | $b^{0++}$ | Seq. | y | $y^{++}$ | y* | $y^{*++}$ | $y^{0}$ | $y^{0++}$ | # |
|---|---|---|---|---|---|---|---|---|---|---|---|---|---|---|
| 1 | 72.0444 | 36.5258 | | | | | A | | | | | | | 7 |
| 2 | **173.0921** | 87.0497 | | | 155.0815 | 78.0444 | T | ***910.4516*** | 455.7295 | 893.4251 | 447.2162 | 892.4411 | 446.7242 | 6 |
| 3 | 465.1980 | 233.1026 | 448.1714 | 224.5894 | 447.1874 | 224.0974 | K | ***809.4040*** | 405.2056 | 792.3774 | 396.6923 | 791.3934 | 396.2003 | 5 |
| 4 | **594.2406** | 297.6239 | 577.2140 | 289.1107 | 576.2300 | 288.6186 | E | ***517.2980*** | 259.1527 | 500.2715 | 250.6394 | 499.2875 | 250.1474 | 4 |
| 5 | **722.2992** | 361.6532 | 705.2726 | 353.1399 | 704.2886 | 352.6479 | Q | ***388.2554*** | 194.6314 | 371.2289 | 186.1181 | | | 3 |
| 6 | **835.3832** | 418.1953 | 818.3567 | 409.6820 | 817.3727 | 409.1900 | L | ***260.1969*** | 130.6021 | 243.1703 | 122.0888 | | | 2 |
| 7 | | | | | | | K | ***147.1128*** | 74.0600 | 130.0863 | 65.5468 | | | 1 |

**Figure S6.** Modified peptide and ESI-MS/MS assigned spectrum of $_{539}$ATKEQLK$_{545}$. For clarity, the amino acid numbering used corresponds to the provided in PDB structures as the first 24 amino acids are usually not observed.

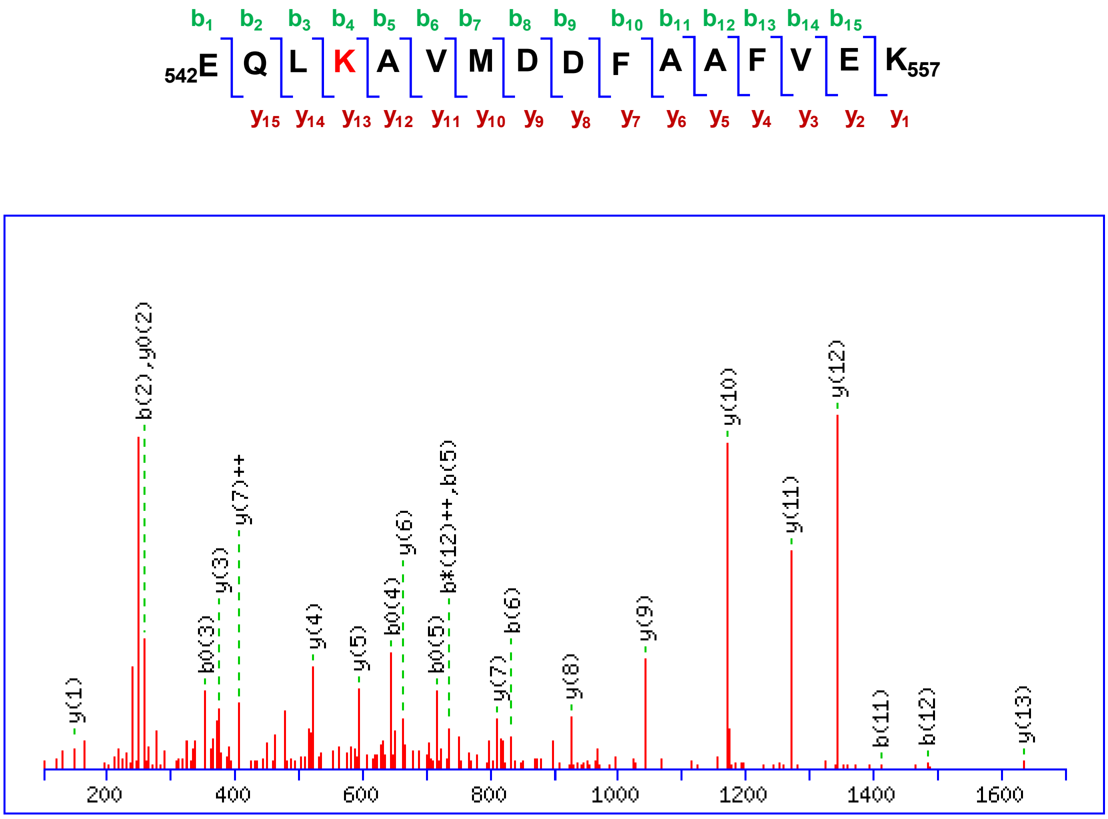


| # | b | $b^{++}$ | b* | $b^{*++}$ | $b^{0}$ | $b^{0++}$ | Seq. | y | $y^{++}$ | y* | $y^{*++}$ | $y^{0}$ | $y^{0++}$ | # |
|---|---|---|---|---|---|---|---|---|---|---|---|---|---|---|
| 1 | 130.0499 | 65.5286 | | | 112.0393 | 56.5233 | E | | | | | | | 16 |
| 2 | **258.1084** | 129.5579 | 241.0819 | 121.0446 | 240.0979 | 120.5526 | Q | 1875.8833 | 938.4453 | 1858.8568 | 929.9320 | 1857.8728 | 929.4400 | 15 |
| 3 | 371.1925 | 186.0999 | 354.1660 | 177.5866 | 353.1819 | 177.0946 | L | 1747.8248 | 874.4160 | 1730.7982 | 865.9027 | 1729.8142 | 865.4107 | 14 |
| 4 | 663.2984 | 332.1529 | 646.2719 | 323.6396 | 645.2879 | 323.1476 | K | ***1634.7407*** | 817.8740 | 1617.7141 | 809.3607 | 1616.7301 | 808.8687 | 13 |
| 5 | **734.3355** | 367.6714 | 717.3090 | 359.1581 | 716.3250 | 358.6661 | A | ***1342.6348*** | 671.8210 | 1325.6082 | 663.3077 | 1324.6242 | 662.8157 | 12 |
| 6 | **833.4040** | 417.2056 | 816.3774 | 408.6923 | 815.3934 | 408.2003 | V | ***1271.5977*** | 636.3025 | 1254.5711 | 627.7892 | 1253.5871 | 627.2972 | 11 |
| 7 | 964.4444 | 482.7259 | 947.4179 | 474.2126 | 946.4339 | 473.7206 | M | ***1172.5292*** | 586.7683 | 1155.5027 | 578.2550 | 1154.5187 | 577.7630 | 10 |
| 8 | 1079.4714 | 540.2393 | 1062.4448 | 531.7261 | 1061.4608 | 531.2341 | D | ***1041.4888*** | 521.2480 | 1024.4622 | 512.7347 | 1023.4782 | 512.2427 | 9 |
| 9 | 1194.4983 | 597.7528 | 1177.4718 | 589.2395 | 1176.4878 | 588.7475 | D | ***926.4618*** | 463.7345 | 909.4353 | 455.2213 | 908.4512 | 454.7293 | 8 |
| 10 | 1341.5667 | 671.2870 | 1324.5402 | 662.7737 | 1323.5562 | 662.2817 | F | ***811.4349*** | 406.2211 | 794.4083 | 397.7078 | 793.4243 | 397.2158 | 7 |
| 11 | **1412.6039** | 706.8056 | 1395.5773 | 698.2923 | 1394.5933 | 697.8003 | A | ***664.3665*** | 332.6869 | 647.3399 | 324.1736 | 646.3559 | 323.6816 | 6 |
| 12 | **1483.6410** | 742.3241 | 1466.6144 | 733.8109 | 1465.6304 | 733.3188 | A | ***593.3293*** | 297.1683 | 576.3028 | 288.6550 | 575.3188 | 288.1630 | 5 |
| 13 | 1630.7094 | 815.8583 | 1613.6828 | 807.3451 | 1612.6988 | 806.8530 | F | ***522.2922*** | 261.6498 | 505.2657 | 253.1365 | 504.2817 | 252.6445 | 4 |
| 14 | 1729.7778 | 865.3925 | 1712.7513 | 856.8793 | 1711.7672 | 856.3873 | V | ***375.2238*** | 188.1155 | 358.1973 | 179.6023 | 357.2132 | 179.1103 | 3 |
| 15 | 1858.8204 | 929.9138 | 1841.7938 | 921.4006 | 1840.8098 | 920.9086 | E | 276.1554 | 138.5813 | 259.1288 | 130.0681 | 258.1448 | 129.5761 | 2 |
| 16 | | | | | | | K | ***147.1128*** | 74.0600 | 130.0863 | 65.5468 | | | 1 |

**Figure S7.** Modified peptide and ESI-MS/MS assigned spectrum of $_{542}$EQLKAVMDDFAAFVEK$_{557}$. For clarity, the amino acid numbering used corresponds to the provided in PDB structures as the first 24 amino acids are usually not observed.

$_{198}$L**K**CASLQK$_{205}$

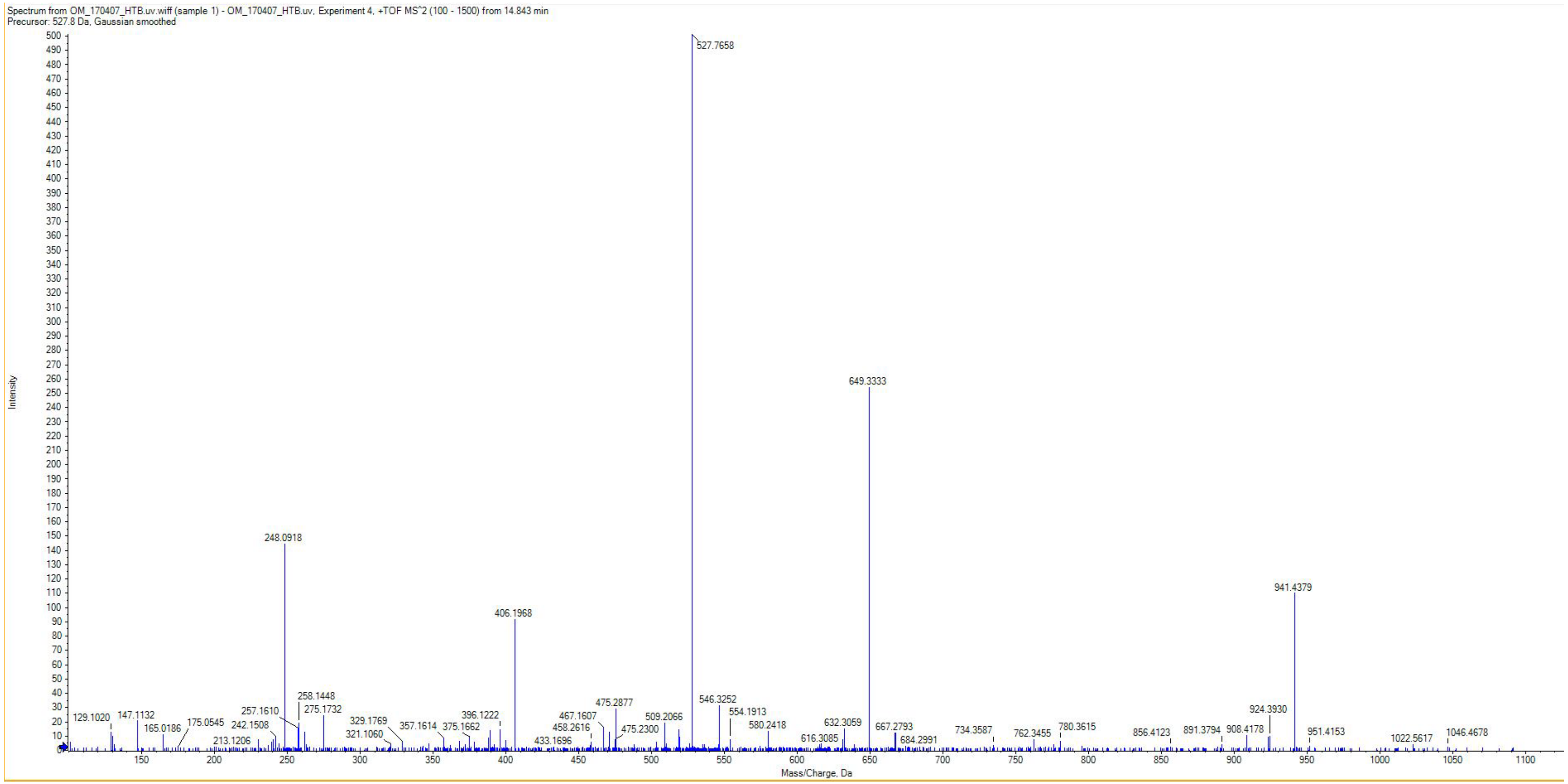


**Figure S8.** Original ESI-HRMS/MS spectrum of the modified peptide $_{198}$L**K**CASLQK$_{205}$

$_{137}$**K**YLYEIAR$_{144}$

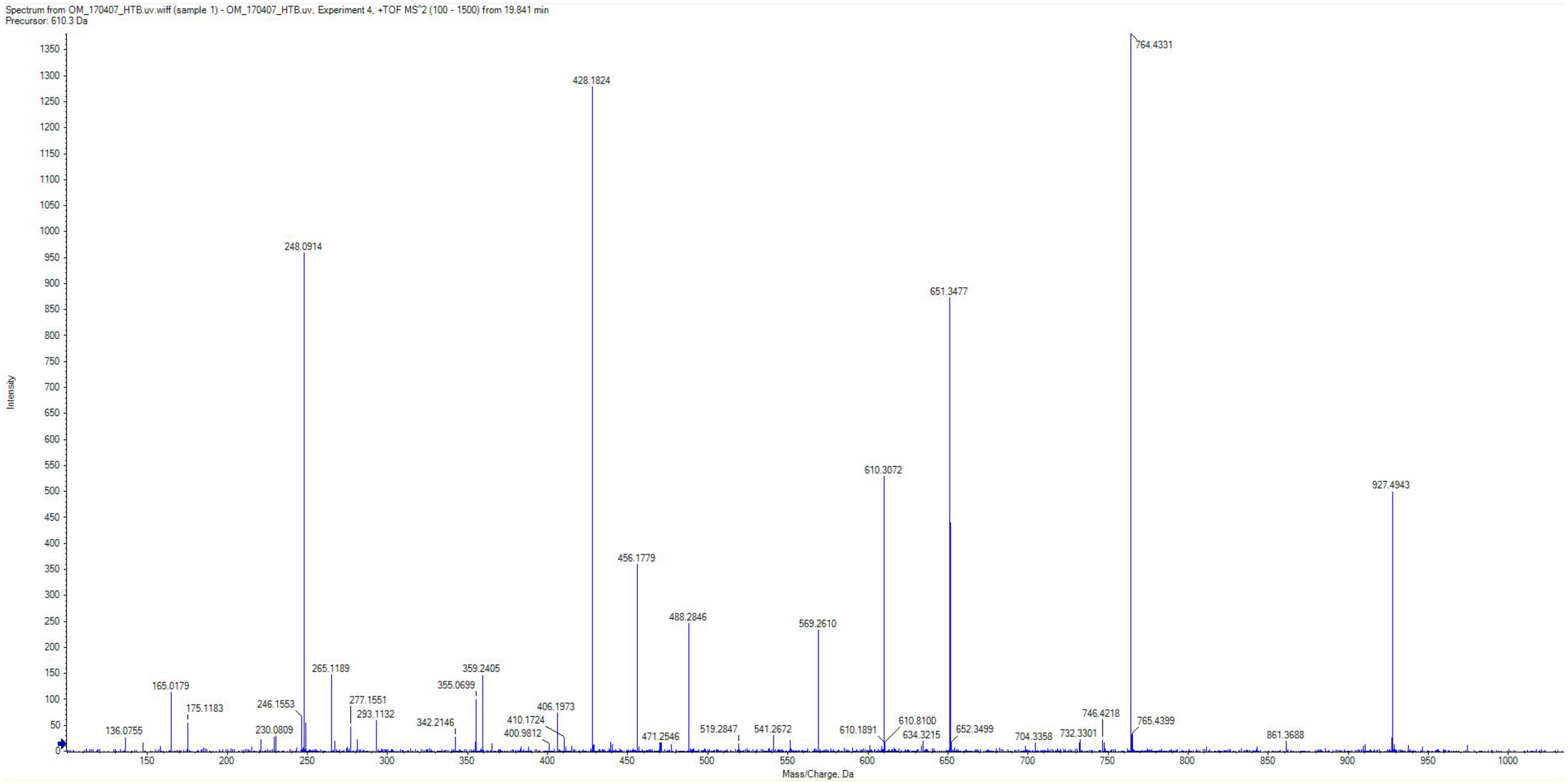


**Figure S9.** Original ESI-HRMS/MS spectrum of the modified peptide $_{137}$**K**YLYEIAR$_{144}$

$_{200}$CASLQ**K**FGER$_{209}$

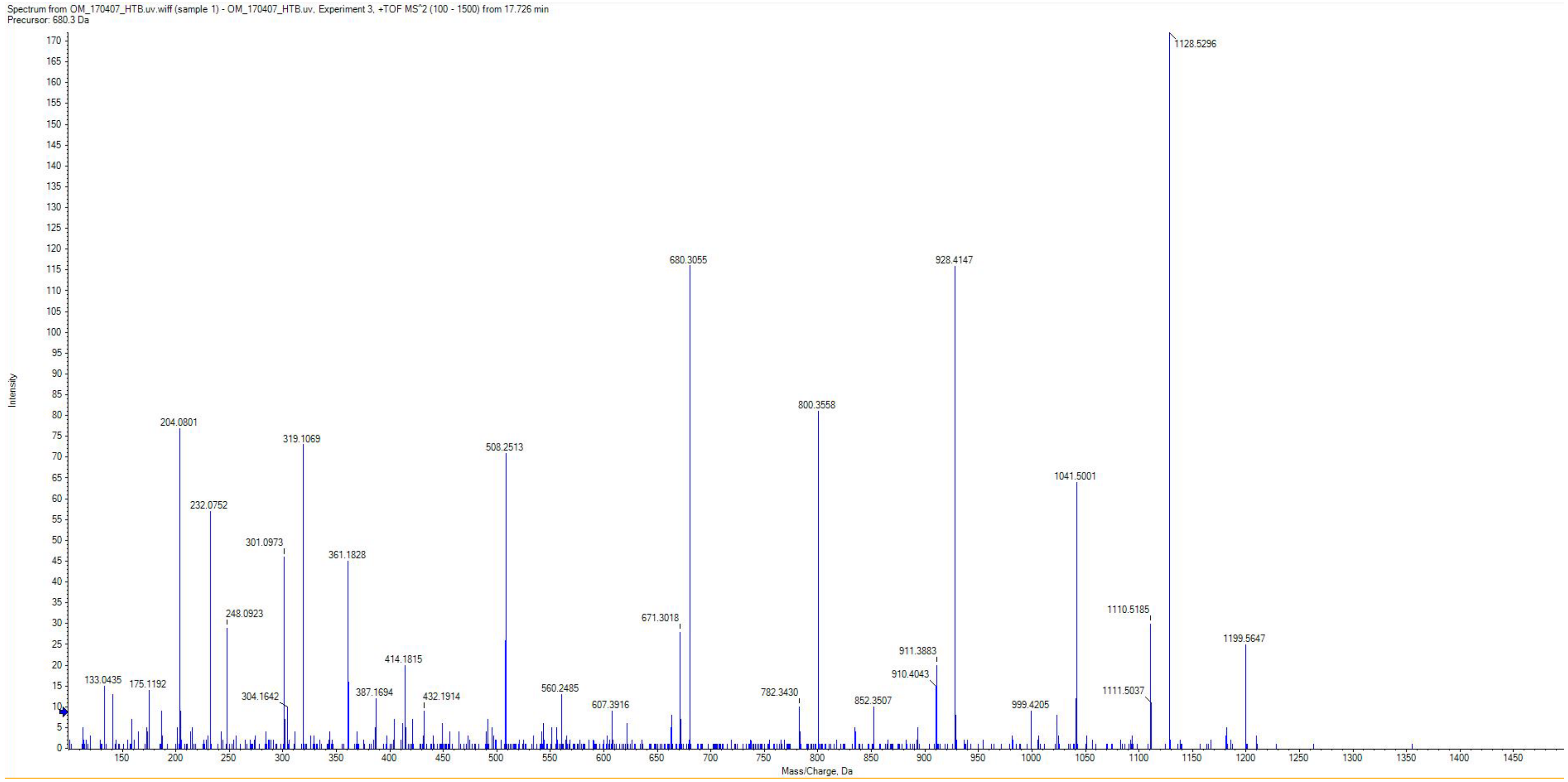


**Figure S10.** Original ESI-HRMS/MS spectrum of the modified peptide $_{200}$CASLQ**K**FGER$_{209}$

$_{349}$LA**K**TYETTLEK$_{359}$

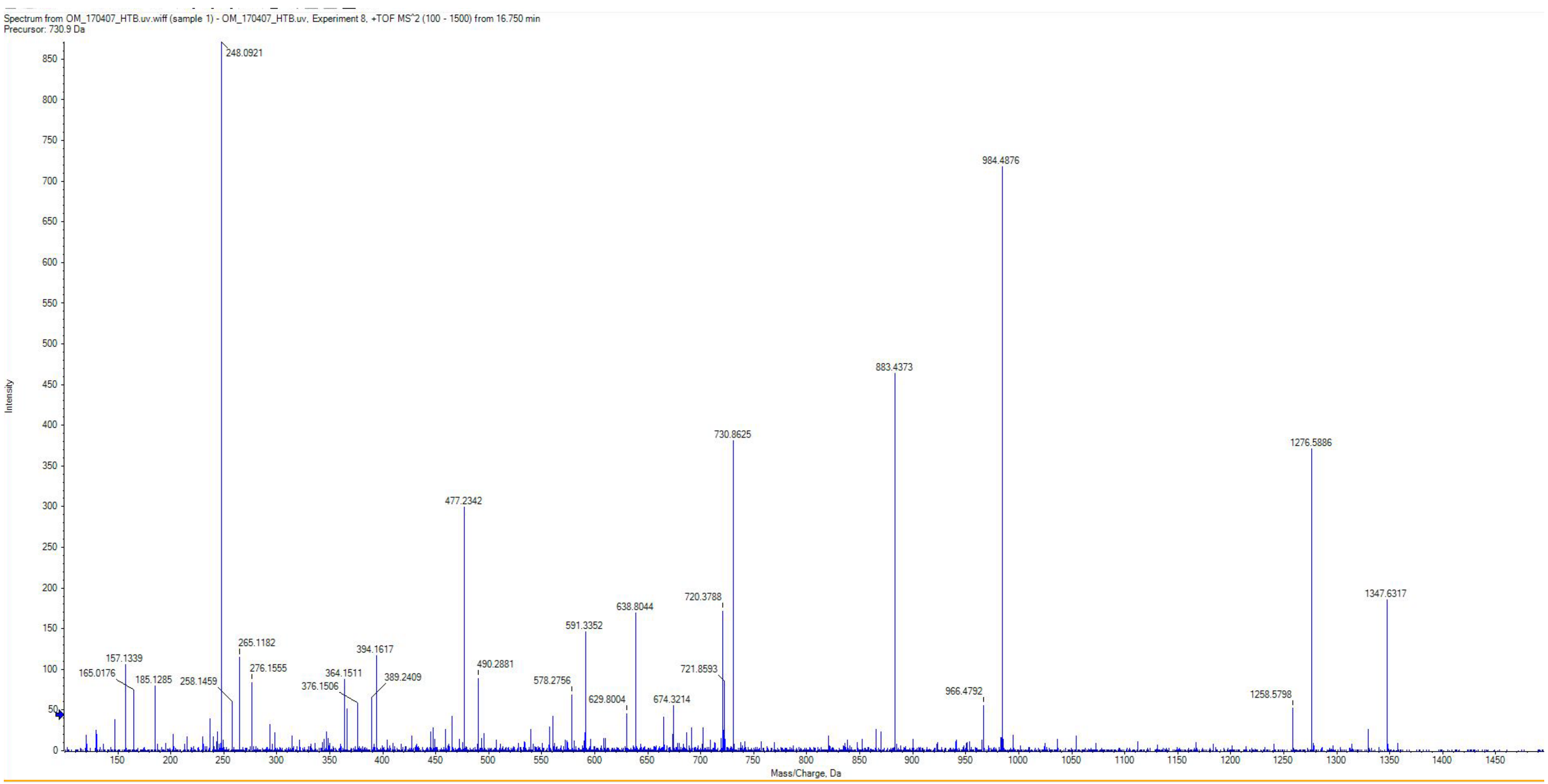


**Figure S11.** Original ESI-HRMS/MS spectrum of the modified peptide $_{349}$LA**K**TYETTLEK$_{359}$

$_{429}$NLG**K**VGSR$_{436}$

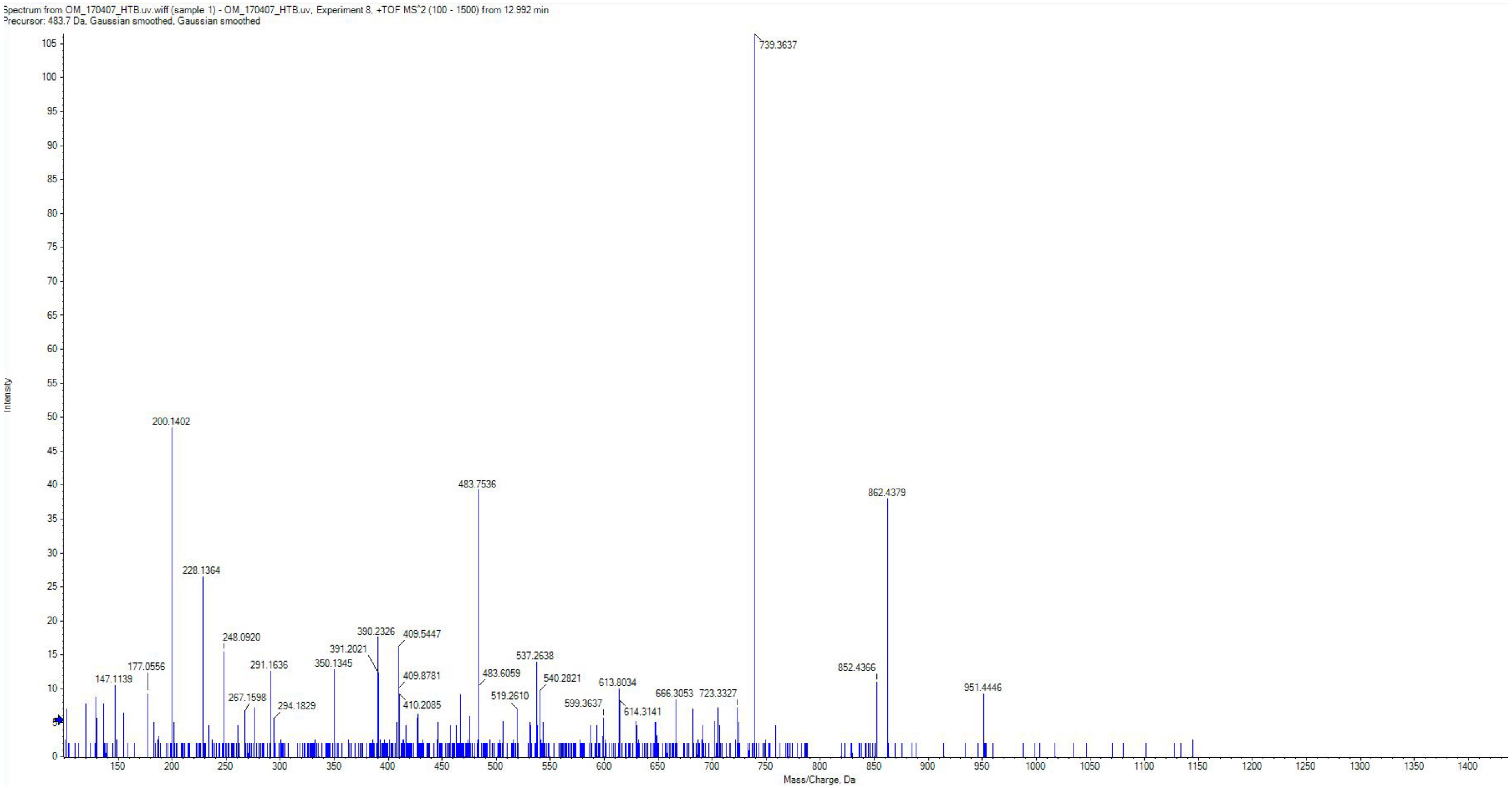


**Figure S12.** Original ESI-HRMS/MS spectrum of the modified peptide $_{429}$NLG**K**VGSR$_{436}$

$_{525}$**K**QTALVELVK$_{534}$

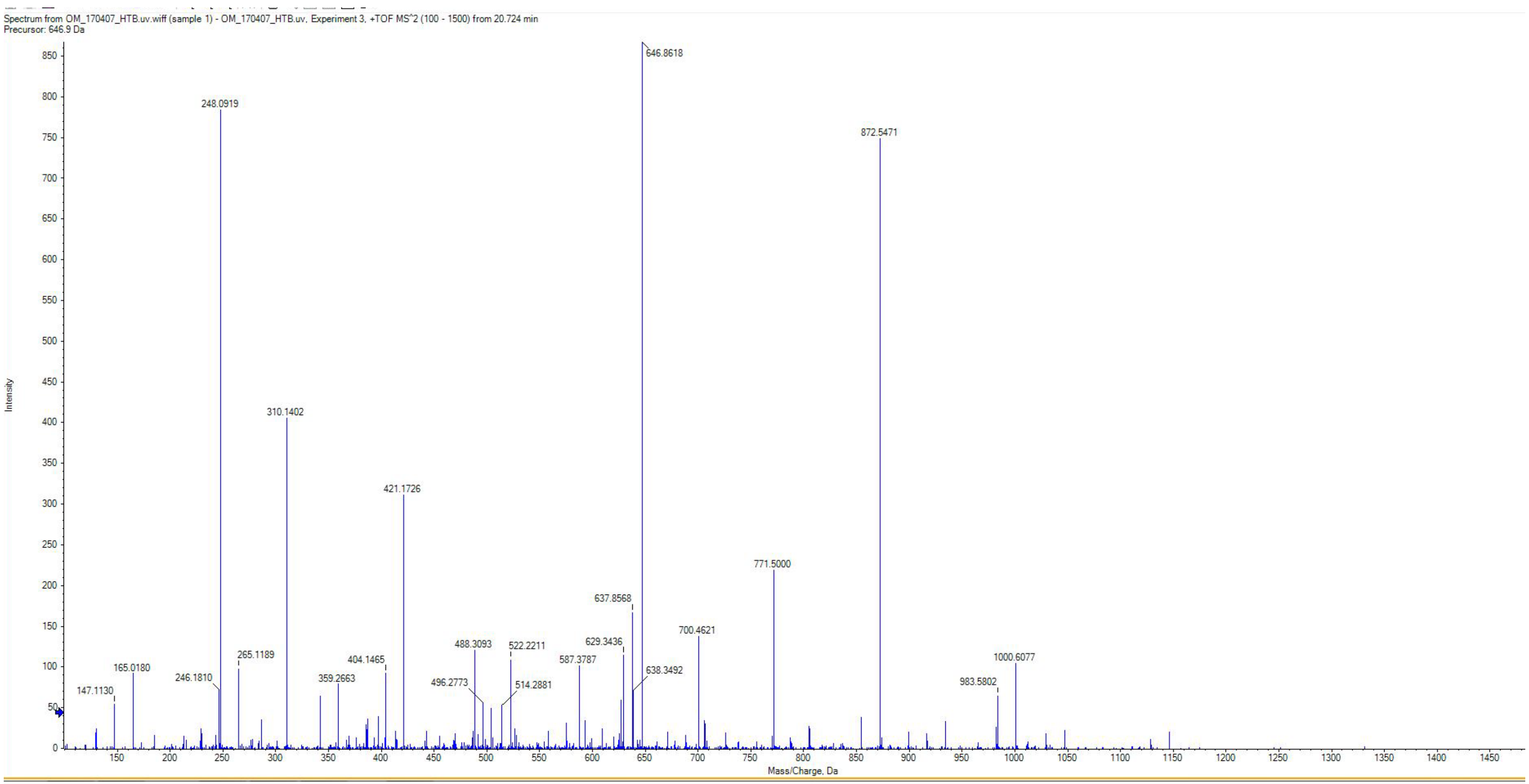


**Figure S13.** Original ESI-HRMS/MS spectrum of the modified peptide $_{525}$**K**QTALVELVK$_{534}$

$_{539}$AT**K**EQLK$_{545}$

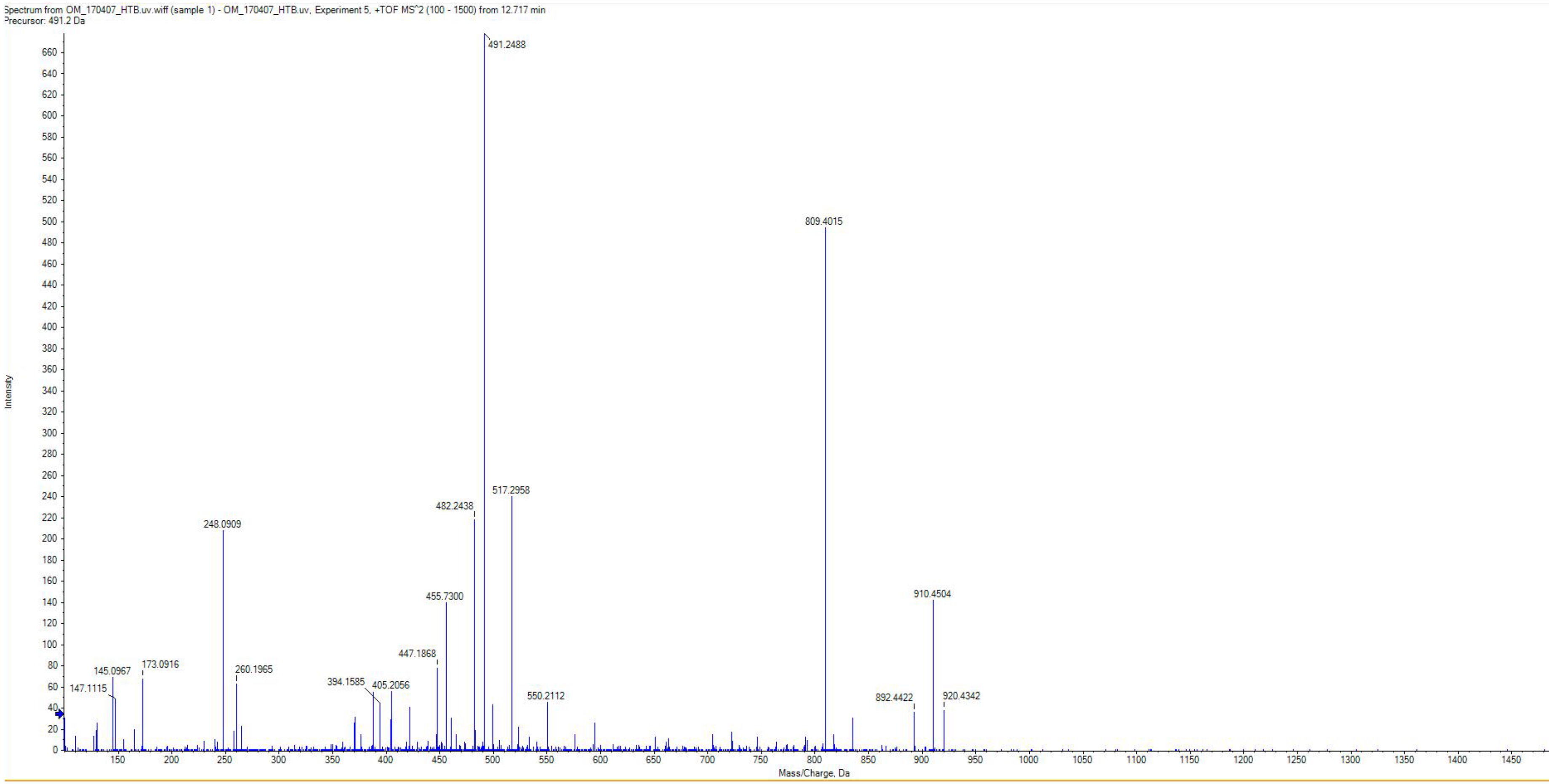


**Figure S14.** Original ESI-HRMS/MS spectrum of the modified peptide $_{539}$AT**K**EQLK$_{545}$

$_{542}$EQL**K**AVMDDFAAFVEK$_{557}$

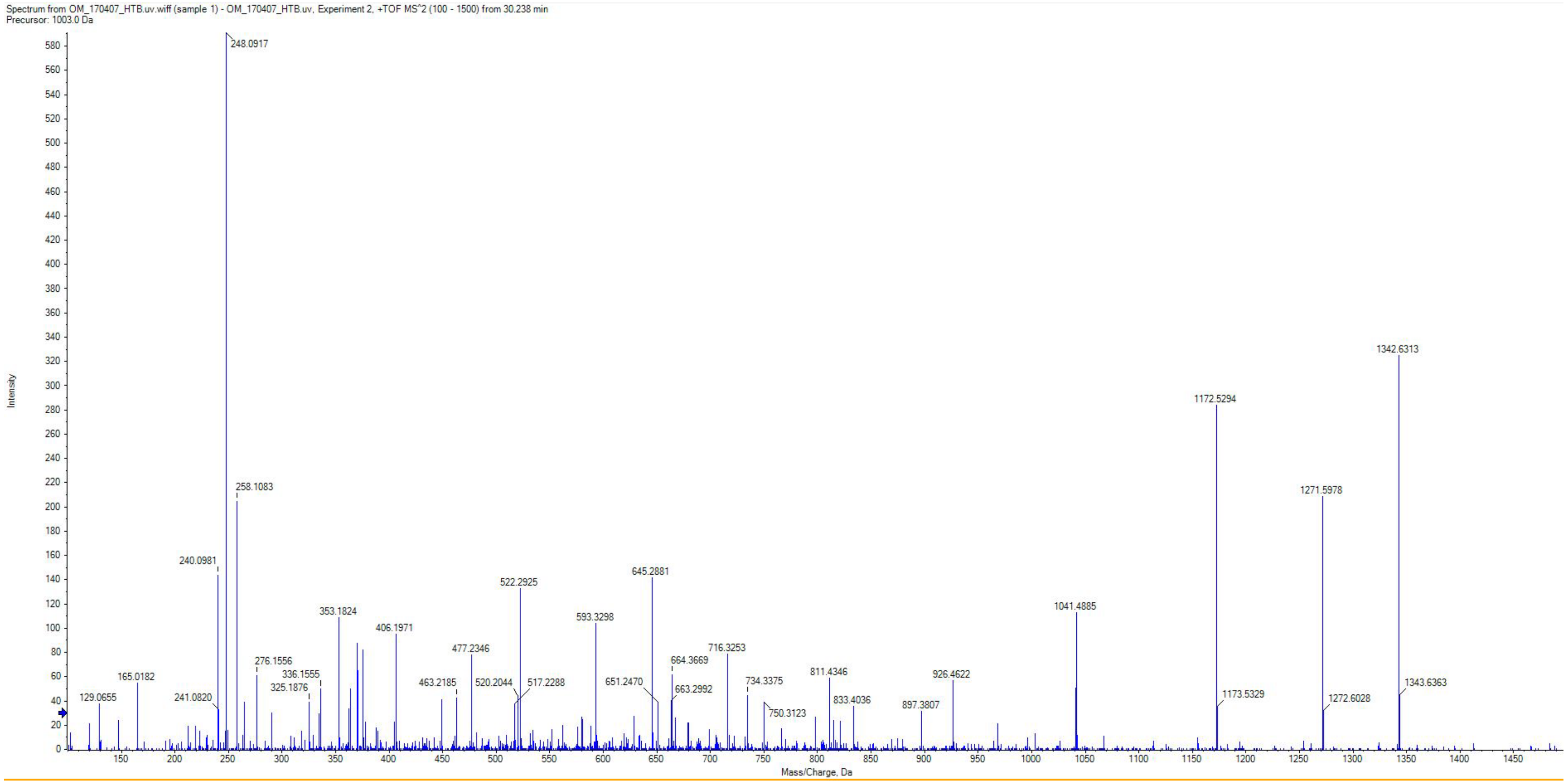


**Figure S15.** Original ESI-HRMS/MS spectrum of the modified peptide $_{542}$EQL**K**AVMDDFAAFVEK$_{557}$

**Table S1.** List of ions detected for $_{198}$L**K**CASLQK$_{205}$

| Mass/Charge | Area | Height | Width | Width at 50% | Resolution | Charge | Monoisotopic | Mass(charge) | Mass/charge (charge) |
|---|---|---|---|---|---|---|---|---|---|
| 101.06819 | 0.02909 | 5.80116 | 0.01986 | 0.00402 | 25159.08309 | 0 | Yes | *100.0604 | *101.0682 |
| 120.07944 | 0.00941 | 2.39365 | 0.02474 | 0.00358 | 33574.84884 | 0 | Yes | *119.0716 | *120.0794 |
| 129.06402 | 0.00976 | 2.39365 | 0.02244 | 0.00371 | 34808.29306 | 0 | Yes | *128.0562 | *129.0640 |
| 129.10202 | 0.14311 | 12.68534 | 0.0513 | 0.01182 | 10925.78652 | 0 | Yes | *128.0942 | *129.1020 |
| 130.08519 | 0.09468 | 10.11586 | 0.06115 | 0.00761 | 17084.01505 | 0 | Yes | *129.0774 | *130.0852 |
| 131.11923 | 0.02295 | 3.98942 | 0.03555 | 0.00374 | 35081.85473 | 2 | Yes | *260.2228 (2) | *131.1192 (2) |
| 147.11318 | 0.22568 | 20.68788 | 0.0445 | 0.00975 | 15091.14779 | 0 | Yes | *146.1053 | *147.1132 |
| 165.01861 | 0.16915 | 11.19782 | 0.058 | 0.01519 | 10862.73496 | 0 | Yes | *164.0108 | *165.0186 |
| 173.09121 | 0.0226 | 2.0277 | 0.04084 | 0.01114 | 15536.71578 | 0 | Yes | *172.0834 | *173.0912 |
| 175.05453 | 0.03409 | 2.61015 | 0.04481 | 0.01785 | 9804.35467 | 0 | Yes | *174.0467 | *175.0545 |
| 200.13577 | 0.01215 | 2.39365 | 0.03194 | 0.00462 | 43345.56014 | 0 | Yes | *199.1279 | *200.1358 |
| 201.12707 | 0.02436 | 2.39446 | 0.04002 | 0.01263 | 15921.18608 | 0 | Yes | *200.1192 | *201.1271 |
| 203.10497 | 0.02448 | 2.0277 | 0.04022 | 0.01207 | 16829.92656 | 0 | Yes | *202.0971 | *203.1050 |
| 213.12058 | 0.0209 | 2.60962 | 0.03296 | 0.00796 | 26774.82789 | 0 | Yes | *212.1128 | *213.1206 |
| 222.1298 | 0.03413 | 2.0277 | 0.05047 | 0.0207 | 10729.39564 | 0 | Yes | *221.1220 | *222.1298 |
| 230.0839 | 0.09118 | 7.72141 | 0.06849 | 0.00714 | 32213.75175 | 1 | Yes | *229.0761 (1) | *230.0839 (1) |
| 231.09234 | 0.04352 | 2.0277 | 0.06864 | 0.03794 | 6091.08489 | 1 | No | 230.0845 (1) | 231.0923 (1) |
| 237.13324 | 0.03086 | 3.98996 | 0.04346 | 0.00519 | 45659.45915 | 0 | Yes | *236.1254 | *237.1332 |
| 239.14758 | 0.09739 | 6.1259 | 0.08292 | 0.0128 | 18677.13642 | 0 | Yes | *238.1398 | *239.1476 |
| 240.13435 | 0.10203 | 7.93737 | 0.05248 | 0.00986 | 24352.59644 | 0 | Yes | *239.1265 | *240.1343 |
| 242.15083 | 0.13809 | 9.59911 | 0.05709 | 0.01186 | 20417.73659 | 0 | Yes | *241.1430 | *242.1508 |
| 244.07548 | 0.07603 | 4.63785 | 0.07495 | 0.01348 | 18112.82206 | 1 | Yes | *243.0677 (1) | *244.0755 (1) |
| 247.31324 | 0.02701 | 2.39365 | 0.04882 | 0.01845 | 13407.10701 | 1 | Yes | *246.3054 (1) | *247.3132 (1) |
| 248.09177 | 2.5475 | 144.89318 | 0.09779 | 0.01547 | 16041.77856 | 0 | Yes | *247.0839 | *248.0918 |
| 254.14667 | 0.04107 | 2.39365 | 0.05849 | 0.02683 | 9472.15496 | 1 | Yes | *253.1388 (1) | *254.1467 (1) |
| 257.16099 | 0.27084 | 15.94339 | 0.06336 | 0.01645 | 15635.32342 | 0 | Yes | *256.1532 | *257.1610 |
| 258.14482 | 0.30355 | 19.17827 | 0.06348 | 0.01457 | 17717.36058 | 1 | Yes | *257.1370 (1) | *258.1448 (1) |
| 262.0864 | 0.18537 | 12.684 | 0.05939 | 0.01085 | 24158.4675 | 1 | Yes | *261.0786 (1) | *262.0864 (1) |
| 263.09995 | 0.05572 | 4.20592 | 0.05493 | 0.0194 | 13560.06387 | 1 | No | 262.0921 (1) | 263.1000 (1) |
| 265.1173 | 0.06525 | 2.82638 | 0.05514 | 0.03008 | 8812.29556 | 0 | Yes | *264.1095 | *265.1173 |
| 272.16395 | 0.06612 | 2.02823 | 0.07914 | 0.04619 | 5892.19234 | 0 | Yes | *271.1561 | *272.1639 |
| 275.17323 | 0.45111 | 24.78823 | 0.09831 | 0.0132 | 20848.75392 | 1 | Yes | *274.1654 (1) | *275.1732 (1) |
| 289.19774 | 0.02434 | 2.60962 | 0.04319 | 0.00927 | 31189.679 | 0 | Yes | *288.1899 | *289.1977 |
| 293.11066 | 0.04901 | 2.02823 | 0.06281 | 0.02861 | 10244.61579 | 2 | Yes | *584.2057 (2) | *293.1107 (2) |
| 311.17782 | 0.04545 | 2.60962 | 0.0896 | 0.05865 | 5305.79187 | 1 | Yes | *310.1700 (1) | *311.1778 (1) |
| 312.15325 | 0.04046 | 2.0277 | 0.05983 | 0.02454 | 12719.178 | 1 | No | 311.1454 (1) | 312.1532 (1) |
| 320.17078 | 0.02561 | 2.39419 | 0.05049 | 0.01448 | 22111.40542 | 0 | Yes | *319.1629 | *320.1708 |
| 321.10597 | 0.08207 | 4.5304 | 0.06574 | 0.01535 | 20920.3068 | 0 | Yes | *320.0981 | *321.1060 |
| 329.17693 | 0.19736 | 6.12724 | 0.1024 | 0.03933 | 8370.39219 | 1 | Yes | *328.1691 (1) | *329.1769 (1) |
| 341.17571 | 0.01586 | 2.39365 | 0.0417 | 0.00603 | 56594.38526 | 0 | Yes | *340.1679 | *341.1757 |
| 343.16718 | 0.03182 | 2.7176 | 0.04705 | 0.01116 | 30736.11606 | 0 | Yes | *342.1594 | *343.1672 |
| 347.17483 | 0.10667 | 4.85328 | 0.08413 | 0.01446 | 24009.07958 | 0 | Yes | *346.1670 | *347.1748 |
| 353.2236 | 0.04842 | 2.61069 | 0.06364 | 0.02402 | 14705.92523 | 1 | Yes | *352.2158 (1) | *353.2236 (1) |
| 357.16137 | 0.28673 | 9.12731 | 0.08533 | 0.02379 | 15015.97428 | 1 | Yes | *356.1535 (1) | *357.1614 (1) |
| 358.17172 | 0.01625 | 2.39365 | 0.04273 | 0.00618 | 57986.92779 | 1 | No | 357.1639 (1) | 358.1717 (1) |
| 362.12899 | 0.04903 | 3.98996 | 0.06444 | 0.00642 | 56424.10274 | 0 | Yes | *361.1212 | *362.1290 |
| 368.13326 | 0.13182 | 6.12644 | 0.08663 | 0.02459 | 14972.0563 | 1 | Yes | *367.1254 (1) | *368.1333 (1) |
| 371.21694 | 0.02758 | 2.39419 | 0.05437 | 0.01559 | 23808.79781 | 3 | No | 1110.6273 (3) | 371.2169 (3) |
| 372.12129 | 0.03865 | 3.98942 | 0.07077 | 0.0063 | 59106.20454 | 0 | Yes | *371.1135 | *372.1213 |
| 375.16623 | 0.27169 | 9.4938 | 0.09839 | 0.02522 | 14875.76579 | 1 | Yes | *374.1584 (1) | *375.1662 (1) |
| 376.19414 | 0.02776 | 2.39365 | 0.06021 | 0.02117 | 17771.81955 | 1 | No | 375.1863 (1) | 376.1941 (1) |
| 378.2049 | 0.13361 | 6.12644 | 0.08232 | 0.02162 | 17496.6941 | 0 | Yes | *377.1971 | *378.2049 |

| | | | | | | | | | |
|---|---|---|---|---|---|---|---|---|---|
| 388.25705 | 0.31587 | 8.91242 | 0.10009 | 0.033 | 11765.25397 | 1 | Yes | *387.2492 (1) | *388.2570 (1) |
| 389.1719 | 0.37836 | 14.24097 | 0.08907 | 0.02619 | 14859.65088 | 1 | Yes | *388.1641 (1) | *389.1719 (1) |
| 390.14663 | 0.03392 | 2.0277 | 0.04459 | 0.01673 | 23325.80711 | 2 | Yes | *778.2776 (2) | *390.1466 (2) |
| 390.6875 | 0.01697 | 2.39365 | 0.04462 | 0.00645 | 60561.89932 | 0 | Yes | *389.6797 | *390.6875 |
| 392.19726 | 0.08504 | 4.3139 | 0.07265 | 0.02119 | 18511.80967 | 2 | Yes | *782.3789 (2) | *392.1973 (2) |
| 396.12224 | 0.31335 | 14.66996 | 0.08425 | 0.01837 | 21558.61604 | 1 | Yes | *395.1144 (1) | *396.1222 (1) |
| 400.22173 | 0.06299 | 7.18096 | 0.07339 | 0.00653 | 61297.27889 | 0 | Yes | *399.2139 | *400.2217 |
| 404.15295 | 0.02877 | 2.60962 | 0.05106 | 0.01096 | 36871.29263 | 0 | Yes | *403.1451 | *404.1529 |
| 406.0942 | 0.02884 | 2.60962 | 0.05118 | 0.01099 | 36959.62189 | 0 | Yes | *405.0864 | *406.0942 |
| 406.1968 | 2.1289 | 91.58046 | 0.10238 | 0.02016 | 20149.18765 | 0 | Yes | *405.1890 | *406.1968 |
| 414.17872 | 0.01748 | 2.39365 | 0.04595 | 0.00664 | 62356.0786 | 0 | Yes | *413.1709 | *414.1787 |
| 422.23497 | 0.01765 | 2.39365 | 0.04639 | 0.00671 | 62959.61458 | 3 | Yes | *1263.6814 (3) | *422.2350 (3) |
| 424.13038 | 0.04716 | 2.02823 | 0.06393 | 0.02325 | 18242.94615 | 0 | Yes | *423.1225 | *424.1304 |
| 433.1696 | 0.05362 | 2.61015 | 0.07635 | 0.03247 | 13339.87893 | 0 | Yes | *432.1618 | *433.1696 |
| 449.15263 | 0.0546 | 2.60962 | 0.10765 | 0.06448 | 6965.58672 | 1 | Yes | *448.1448 (1) | *449.1526 (1) |
| 452.69859 | 0.01827 | 2.39365 | 0.04803 | 0.00694 | 65191.31421 | 0 | Yes | *451.6908 | *452.6986 |
| 457.22361 | 0.04897 | 2.0277 | 0.08448 | 0.04177 | 10946.50148 | 0 | Yes | *456.2158 | *457.2236 |
| 458.20314 | 0.03064 | 3.98942 | 0.04228 | 0.00699 | 65586.46599 | 0 | Yes | *457.1953 | *458.2031 |
| 458.2616 | 0.11643 | 6.1259 | 0.08458 | 0.01159 | 39531.27793 | 0 | Yes | *457.2538 | *458.2616 |
| 461.1823 | 0.03074 | 2.60962 | 0.05454 | 0.01171 | 39386.79691 | 0 | Yes | *460.1745 | *461.1823 |
| 467.16067 | 0.4331 | 15.94366 | 0.10369 | 0.0269 | 17364.97708 | 1 | Yes | *466.1528 (1) | *467.1607 (1) |
| 470.25848 | 0.03104 | 2.39419 | 0.05508 | 0.01755 | 26797.44286 | 0 | Yes | *469.2506 | *470.2585 |
| 471.22806 | 0.31692 | 13.0521 | 0.08576 | 0.02461 | 19149.48634 | 0 | Yes | *470.2202 | *471.2281 |
| 474.2622 | 0.04987 | 2.0277 | 0.07375 | 0.03025 | 15677.69125 | 2 | No | 946.5087 (2) | 474.2622 (2) |
| 475.22999 | 0.16369 | 7.93898 | 0.06152 | 0.01714 | 27719.92024 | 0 | Yes | *474.2222 | *475.2300 |
| 475.28775 | 0.84417 | 29.17265 | 0.10459 | 0.02804 | 16948.00144 | 0 | Yes | *474.2799 | *475.2877 |
| 484.24806 | 0.0189 | 2.39365 | 0.04968 | 0.00718 | 67424.74317 | 0 | Yes | *483.2402 | *484.2481 |
| 487.75936 | 0.05058 | 2.0277 | 0.07479 | 0.03068 | 15899.29646 | 2 | Yes | *973.5031 (2) | *487.7594 (2) |
| 488.25558 | 0.0759 | 4.3139 | 0.06859 | 0.01484 | 32899.82541 | 2 | No | 974.4955 (2) | 488.2556 (2) |
| 499.28509 | 0.08315 | 2.61042 | 0.08828 | 0.04475 | 11157.009 | 0 | Yes | *498.2773 | *499.2851 |
| 503.22901 | 0.19907 | 5.91074 | 0.12661 | 0.02703 | 18617.35917 | 1 | Yes | *502.2212 (1) | *503.2290 (1) |
| 509.20662 | 0.68472 | 19.50383 | 0.12736 | 0.02836 | 17956.04086 | 1 | Yes | *508.1988 (1) | *509.2066 (1) |
| 509.85887 | 0.01939 | 2.39365 | 0.05098 | 0.00737 | 69184.76821 | 0 | Yes | *508.8510 | *509.8589 |
| 510.26548 | 0.0776 | 4.52933 | 0.07012 | 0.01404 | 36330.98939 | 0 | Yes | *509.2577 | *510.2655 |
| 517.22122 | 0.03255 | 2.39419 | 0.06418 | 0.0184 | 28103.72456 | 1 | Yes | *516.2134 (1) | *517.2212 (1) |
| 518.76032 | 0.2608 | 14.86169 | 0.10927 | 0.01291 | 40192.90798 | 2 | Yes | *1035.5050 (2) | *518.7603 (2) |
| 519.25473 | 0.19569 | 9.27597 | 0.07717 | 0.01978 | 26251.35034 | 2 | No | 1036.4938 (2) | 519.2547 (2) |
| 523.78651 | 0.01965 | 2.39365 | 0.05167 | 0.00747 | 70123.36176 | 0 | Yes | *522.7787 | *523.7865 |
| 526.08305 | 0.09262 | 4.42215 | 0.07767 | 0.0236 | 22289.26949 | 3 | Yes | *1575.2257 (3) | *526.0831 (3) |
| 526.19304 | 0.0598 | 2.60962 | 0.10358 | 0.05685 | 9255.96644 | 3 | Yes | *1575.5556 (3) | *526.1930 (3) |
| 527.51722 | 0.11835 | 2.39365 | 0.12315 | 0.08403 | 6278.01679 | 2 | No | 1053.0188 (2) | 527.5172 (2) |
| 527.61446 | 0.05988 | 3.98996 | 0.07779 | 0.00775 | 68107.30461 | 3 | Yes | *1579.8199 (3) | *527.6145 (3) |
| 527.70035 | 0.03288 | 2.60962 | 0.03241 | 0.01287 | 41014.03469 | 0 | Yes | *526.6925 | *527.7003 |
| 527.76584 | 15.42373 | 500.92914 | 0.17505 | 0.02848 | 18532.42412 | 0 | Yes | *526.7580 | *527.7658 |
| 527.94512 | 0.07893 | 2.60962 | 0.11023 | 0.05694 | 9271.44544 | 3 | No | 1580.8119 (3) | 527.9451 (3) |
| 528.28357 | 0.13159 | 2.82612 | 0.12324 | 0.07651 | 6904.97308 | 3 | No | 1581.8272 (3) | 528.2836 (3) |
| 529.30096 | 0.08562 | 2.39419 | 0.11037 | 0.06281 | 8426.62497 | 1 | Yes | *528.2931 (1) | *529.3010 (1) |
| 535.22379 | 0.0596 | 4.20539 | 0.07182 | 0.0104 | 51449.40926 | 0 | Yes | *534.2160 | *535.2238 |
| 536.1767 | 0.14512 | 4.31417 | 0.09148 | 0.03794 | 14132.00261 | 1 | Yes | *535.1689 (1) | *536.1767 (1) |
| 539.27213 | 0.03324 | 2.39365 | 0.07864 | 0.0319 | 16906.489 | 0 | Yes | *538.2643 | *539.2721 |
| 546.26522 | 0.05352 | 4.20592 | 0.03957 | 0.01547 | 35319.10798 | 0 | Yes | *545.2574 | *546.2652 |
| 546.32523 | 1.24523 | 31.69825 | 0.10554 | 0.03527 | 15490.22071 | 0 | Yes | *545.3174 | *546.3252 |
| 551.20895 | 0.0336 | 2.39365 | 0.08613 | 0.03887 | 14179.43346 | 2 | No | 1100.4022 (2) | 551.2090 (2) |
| 552.25612 | 0.03364 | 2.39419 | 0.06632 | 0.01902 | 29039.84988 | 2 | No | 1102.4966 (2) | 552.2561 (2) |
| 554.19131 | 0.16173 | 8.04615 | 0.07972 | 0.02084 | 26589.76787 | 0 | Yes | *553.1835 | *554.1913 |

| | | | | | | | | | |
|---|---|---|---|---|---|---|---|---|---|
| 561.18356 | 0.04747 | 2.39419 | 0.07354 | 0.0265 | 21179.30398 | 0 | Yes | *560.1757 | *561.1836 |
| 574.20389 | 0.04116 | 2.0277 | 0.06086 | 0.02029 | 28298.02842 | 0 | Yes | *573.1961 | *574.2039 |
| 574.27331 | 0.08232 | 2.93356 | 0.1082 | 0.05827 | 9855.38576 | 0 | Yes | *573.2655 | *574.2733 |
| 578.23733 | 0.10325 | 2.60962 | 0.1425 | 0.09322 | 6203.08167 | 2 | Yes | *1154.4590 (2) | *578.2373 (2) |
| 580.2418 | 0.48268 | 13.27048 | 0.12916 | 0.04011 | 14466.21783 | 1 | Yes | *579.2340 (1) | *580.2418 (1) |
| 588.31656 | 0.10415 | 2.60962 | 0.12321 | 0.07453 | 7893.4683 | 1 | Yes | *587.3087 (1) | *588.3166 (1) |
| 590.30107 | 0.02087 | 2.39365 | 0.05485 | 0.00793 | 74442.79435 | 0 | Yes | *589.2932 | *590.3011 |
| 596.36042 | 0.05592 | 2.02823 | 0.07581 | 0.02757 | 21632.16188 | 1 | Yes | *595.3526 (1) | *596.3604 (1) |
| 614.30044 | 0.05676 | 2.60962 | 0.09093 | 0.04118 | 14918.44699 | 0 | Yes | *613.2926 | *614.3004 |
| 615.27566 | 0.07101 | 3.99076 | 0.091 | 0.01967 | 31282.83047 | 1 | Yes | *614.2678 (1) | *615.2757 (1) |
| 616.30849 | 0.09239 | 4.52933 | 0.09808 | 0.01496 | 41190.60892 | 1 | Yes | *615.3007 (1) | *616.3085 (1) |
| 621.27718 | 0.09989 | 2.61015 | 0.11254 | 0.06221 | 9987.40146 | 1 | Yes | *620.2694 (1) | *621.2772 (1) |
| 622.24613 | 0.06427 | 2.39365 | 0.13375 | 0.08354 | 7448.3898 | 1 | No | 621.2383 (1) | 622.2461 (1) |
| 631.2495 | 0.03481 | 2.39419 | 0.04963 | 0.02033 | 31047.46574 | 2 | No | 1260.4833 (2) | 631.2495 (2) |
| 631.3197 | 0.24455 | 7.72301 | 0.10636 | 0.03947 | 15996.55604 | 0 | Yes | *630.3119 | *631.3197 |
| 632.30589 | 0.47508 | 15.18591 | 0.15612 | 0.0347 | 18219.87811 | 1 | Yes | *631.2981 (1) | *632.3059 (1) |
| 639.2877 | 0.13752 | 4.42215 | 0.10703 | 0.04293 | 14892.66417 | 1 | Yes | *638.2799 (1) | *639.2877 (1) |
| 648.31486 | 0.07289 | 2.0277 | 0.1006 | 0.04974 | 13034.15296 | 0 | Yes | *647.3070 | *648.3149 |
| 649.33326 | 11.0365 | 254.40488 | 0.2373 | 0.03631 | 17882.38637 | 0 | Yes | *648.3254 | *649.3333 |
| 662.32181 | 0.0221 | 2.39365 | 0.0581 | 0.0084 | 78853.44907 | 0 | Yes | *661.3140 | *662.3218 |
| 667.27932 | 0.43628 | 12.57816 | 0.15308 | 0.02991 | 22311.23414 | 0 | Yes | *666.2715 | *667.2793 |
| 674.28536 | 0.07433 | 2.71813 | 0.10259 | 0.04938 | 13653.8293 | 1 | Yes | *673.2775 (1) | *674.2854 (1) |
| 675.8605 | 0.06698 | 2.39365 | 0.10271 | 0.05038 | 13414.09332 | 2 | Yes | *1349.7053 (2) | *675.8605 (2) |
| 676.36139 | 0.05211 | 2.39472 | 0.08807 | 0.0336 | 20129.21222 | 2 | No | 1350.7071 (2) | 676.3614 (2) |
| 676.88257 | 0.02234 | 2.39365 | 0.05874 | 0.00849 | 79715.51979 | 2 | No | 1351.7495 (2) | 676.8826 (2) |
| 684.29911 | 0.06739 | 2.82612 | 0.0812 | 0.02619 | 26126.29007 | 0 | Yes | *683.2913 | *684.2991 |
| 686.27597 | 0.0225 | 2.39365 | 0.05914 | 0.00855 | 80266.74355 | 2 | Yes | *1370.5363 (2) | *686.2760 (2) |
| 691.30988 | 0.09785 | 2.7184 | 0.11872 | 0.06484 | 10661.18092 | 0 | Yes | *690.3020 | *691.3099 |
| 728.36271 | 0.05408 | 2.60962 | 0.09139 | 0.04403 | 16540.63078 | 1 | Yes | *727.3549 (1) | *728.3627 (1) |
| 732.28821 | 0.02324 | 2.39365 | 0.06109 | 0.00883 | 82913.91458 | 0 | Yes | *731.2804 | *732.2882 |
| 734.35868 | 0.1086 | 3.98942 | 0.15295 | 0.04431 | 16574.47687 | 0 | Yes | *733.3509 | *734.3587 |
| 752.3799 | 0.09422 | 2.61015 | 0.10837 | 0.05297 | 14202.71797 | 1 | Yes | *751.3721 (1) | *752.3799 (1) |
| 758.45935 | 0.02365 | 2.39365 | 0.06217 | 0.00899 | 84382.5476 | 0 | Yes | *757.4515 | *758.4594 |
| 760.29163 | 0.03947 | 2.60962 | 0.07003 | 0.01503 | 50571.54274 | 0 | Yes | *759.2838 | *760.2916 |
| 762.34552 | 0.30035 | 7.72328 | 0.15584 | 0.03917 | 19461.0776 | 1 | Yes | *761.3377 (1) | *762.3455 (1) |
| 767.24349 | 0.02379 | 2.39365 | 0.05472 | 0.00904 | 84869.78617 | 0 | Yes | *766.2357 | *767.2435 |
| 770.23237 | 0.02383 | 2.39365 | 0.06266 | 0.00906 | 85034.93713 | 0 | Yes | *769.2245 | *770.2324 |
| 772.40334 | 0.02387 | 2.39365 | 0.06274 | 0.00907 | 85154.69319 | 0 | Yes | *771.3955 | *772.4033 |
| 775.92097 | 0.07176 | 4.42135 | 0.06289 | 0.01521 | 51016.31905 | 2 | Yes | *1549.8263 (2) | *775.9210 (2) |
| 780.36145 | 0.27189 | 6.66715 | 0.13402 | 0.03548 | 21993.34887 | 0 | Yes | *779.3536 | *780.3615 |
| 795.34417 | 0.20182 | 3.04315 | 0.15121 | 0.08984 | 8853.12978 | 1 | Yes | *794.3363 (1) | *795.3442 (1) |
| 856.41231 | 0.02513 | 2.39365 | 0.06607 | 0.00955 | 89666.08135 | 0 | Yes | *855.4045 | *856.4123 |
| 888.39289 | 0.07679 | 2.39419 | 0.11776 | 0.05614 | 15824.02961 | 0 | Yes | *887.3851 | *888.3929 |
| 891.37936 | 0.05983 | 4.20539 | 0.07583 | 0.0134 | 66500.37994 | 1 | No | 890.3715 (1) | 891.3794 (1) |
| 908.4178 | 0.43139 | 10.33504 | 0.17861 | 0.0426 | 21323.91458 | 1 | Yes | *907.4100 (1) | *908.4178 (1) |
| 923.42823 | 0.33056 | 9.49193 | 0.15436 | 0.02682 | 34424.76669 | 0 | Yes | *922.4204 | *923.4282 |
| 924.39301 | 0.23499 | 9.79192 | 0.11154 | 0.01143 | 80904.33272 | 0 | Yes | *923.3852 | *924.3930 |
| 941.43792 | 6.28003 | 109.98362 | 0.30306 | 0.05313 | 17718.18592 | 0 | Yes | *940.4301 | *941.4379 |
| 942.46625 | 0.04394 | 2.39419 | 0.08663 | 0.02484 | 37936.52668 | 0 | Yes | *941.4584 | *942.4663 |
| 951.4153 | 0.20308 | 3.04262 | 0.1915 | 0.12392 | 7677.60567 | 1 | No | 950.4075 (1) | 951.4153 (1) |
| 1022.56167 | 0.06408 | 3.98942 | 0.09926 | 0.01044 | 97971.77345 | 0 | Yes | *1021.5538 | *1022.5617 |
| 1046.46782 | 0.07408 | 2.60962 | 0.10955 | 0.04653 | 22489.62511 | 0 | Yes | *1045.4600 | *1046.4678 |

**Table S2.** List of ions detected for $_{137}$**KYLYEIAR**$_{144}$

| Mass/Charge | Area | Height | Width | Width at 50% | Resolution | Charge | Monoisotopic | Mass(charge) | Mass/charge (charge) |
|---|---|---|---|---|---|---|---|---|---|
| 136.07549 | 0.27488 | 27 | 0.01975 | 0.0102 | 13334.26761 | 0 | Yes | *135.0677 | *136.0755 |
| 147.00714 | 0.17108 | 17 | 0.02737 | 0.008 | 18366.55549 | 0 | Yes | *145.9993 | *147.0071 |
| 165.01788 | 1.52074 | 114 | 0.03625 | 0.01208 | 13656.14822 | 0 | Yes | *164.0101 | *165.0179 |
| 175.11828 | 0.67967 | 55 | 0.04108 | 0.01188 | 14746.45727 | 0 | Yes | *174.1105 | *175.1183 |
| 221.08497 | 0.29792 | 23 | 0.03357 | 0.0111 | 19913.94257 | 0 | Yes | *220.0771 | *221.0850 |
| 229.12871 | 0.42717 | 28 | 0.0299 | 0.01417 | 16165.17915 | 0 | Yes | *228.1209 | *229.1287 |
| 230.08089 | 0.31676 | 29 | 0.03424 | 0.00999 | 23035.88515 | 0 | Yes | *229.0731 | *230.0809 |
| 246.1553 | 1.29727 | 68 | 0.07527 | 0.01541 | 15978.57383 | 0 | Yes | *245.1475 | *246.1553 |
| 248.09138 | 14.74161 | 959 | 0.10223 | 0.01365 | 18179.9512 | 0 | Yes | *247.0836 | *248.0914 |
| 249.15997 | 0.95994 | 55 | 0.04455 | 0.01525 | 16339.87726 | 0 | Yes | *248.1521 | *249.1600 |
| 265.11888 | 2.10908 | 148 | 0.05054 | 0.01207 | 21960.74696 | 0 | Yes | *264.1110 | *265.1189 |
| 266.99851 | 0.25823 | 19 | 0.0415 | 0.01057 | 25248.80119 | 0 | Yes | *265.9907 | *266.9985 |
| 277.15509 | 1.01948 | 47 | 0.06577 | 0.02089 | 13269.38544 | 0 | Yes | *276.1473 | *277.1551 |
| 281.05136 | 0.38321 | 22 | 0.04258 | 0.0163 | 17237.13422 | 0 | Yes | *280.0435 | *281.0514 |
| 293.11323 | 1.03634 | 60 | 0.05315 | 0.0141 | 20792.7927 | 0 | Yes | *292.1054 | *293.1132 |
| 342.21455 | 0.54815 | 27 | 0.05742 | 0.01887 | 18137.1262 | 0 | Yes | *341.2067 | *342.2146 |
| 355.06992 | 2.18554 | 100 | 0.07976 | 0.01882 | 18865.62466 | 0 | Yes | *354.0621 | *355.0699 |
| 359.2405 | 3.13971 | 147 | 0.13371 | 0.01845 | 19475.31476 | 0 | Yes | *358.2327 | *359.2405 |
| 365.15019 | 0.36669 | 15 | 0.05932 | 0.02244 | 16272.99998 | 0 | Yes | *364.1424 | *365.1502 |
| 400.98118 | 0.24864 | 15 | 0.05651 | 0.01589 | 25229.57512 | 0 | Yes | *399.9733 | *400.9812 |
| 406.19728 | 1.8314 | 74 | 0.091 | 0.02292 | 17724.27141 | 0 | Yes | *405.1894 | *406.1973 |
| 410.17239 | 0.69441 | 28 | 0.06858 | 0.02072 | 19797.7703 | 0 | Yes | *409.1646 | *410.1724 |
| 428.18243 | 33.45141 | 1279 | 0.1635 | 0.02357 | 18165.61814 | 0 | Yes | *427.1746 | *428.1824 |
| 429.1847 | 0.46478 | 14 | 0.08185 | 0.02874 | 14931.11343 | 0 | Yes | *428.1769 | *429.1847 |
| 439.15088 | 0.49676 | 18 | 0.06505 | 0.02996 | 14656.36907 | 1 | Yes | *438.1430 (1) | *439.1509 (1) |
| 440.21961 | 0.24868 | 14 | 0.06513 | 0.00791 | 55635.46697 | 1 | Yes | *439.2118 (1) | *440.2196 (1) |
| 456.17795 | 10.70758 | 360 | 0.13863 | 0.02649 | 17218.86276 | 0 | Yes | *455.1701 | *456.1779 |
| 470.27347 | 0.62728 | 17 | 0.05508 | 0.03679 | 12782.18807 | 1 | Yes | *469.2656 (1) | *470.2735 (1) |
| 471.2546 | 0.4564 | 17 | 0.07351 | 0.03814 | 12357.42731 | 1 | No | 470.2468 (1) | 471.2546 (1) |
| 488.28458 | 7.63268 | 246 | 0.11848 | 0.02805 | 17409.43707 | 0 | Yes | *487.2768 | *488.2846 |
| 519.28474 | 0.3344 | 15 | 0.05788 | 0.01833 | 28333.45065 | 2 | Yes | *1036.5538 (2) | *519.2847 (2) |
| 541.26723 | 0.97826 | 31 | 0.07879 | 0.03429 | 15785.7787 | 1 | Yes | *540.2594 (1) | *541.2672 (1) |
| 551.24802 | 0.76196 | 22 | 0.08613 | 0.03423 | 16102.92951 | 1 | Yes | *550.2402 (1) | *551.2480 (1) |
| 569.26102 | 8.40964 | 234 | 0.1414 | 0.03163 | 17999.70228 | 0 | Yes | *568.2532 | *569.2610 |
| 610.18913 | 0.71452 | 18 | 0.12547 | 0.03547 | 17200.89644 | 0 | Yes | *609.1813 | *610.1891 |
| 610.30725 | 18.66998 | 530 | 0.16732 | 0.0328 | 18607.90804 | 0 | Yes | *609.2994 | *610.3072 |
| 610.80999 | 0.54401 | 18 | 0.06277 | 0.03036 | 20116.54054 | 0 | Yes | *609.8022 | *610.8100 |
| 634.32152 | 0.65744 | 19 | 0.10661 | 0.03258 | 19472.18725 | 0 | Yes | *633.3137 | *634.3215 |
| 651.34767 | 35.14677 | 874 | 0.21607 | 0.03595 | 18120.47984 | 0 | Yes | *650.3398 | *651.3477 |
| 652.34995 | 0.66311 | 19 | 0.07929 | 0.04114 | 15855.16858 | 0 | Yes | *651.3421 | *652.3499 |
| 704.33584 | 0.65907 | 17 | 0.09736 | 0.05029 | 14006.48509 | 0 | Yes | *703.3280 | *704.3358 |
| 732.33007 | 0.96606 | 23 | 0.09928 | 0.03899 | 18784.87514 | 0 | Yes | *731.3222 | *732.3301 |
| 746.42182 | 0.78641 | 22 | 0.08481 | 0.0302 | 24718.17289 | 1 | Yes | *745.4140 (1) | *746.4218 (1) |
| 747.41157 | 0.82937 | 17 | 0.10029 | 0.05915 | 12636.17235 | 1 | No | 746.4037 (1) | 747.4116 (1) |
| 764.43313 | 65.21644 | 1381 | 0.19506 | 0.04345 | 17594.67408 | 0 | Yes | *763.4253 | *764.4331 |
| 765.43994 | 1.58883 | 36 | 0.17176 | 0.0387 | 19778.40535 | 0 | Yes | *764.4321 | *765.4399 |
| 861.36881 | 1.04358 | 19 | 0.14908 | 0.05913 | 14568.16973 | 1 | Yes | *860.3610 (1) | *861.3688 (1) |
| 927.4943 | 28.3271 | 500 | 0.24065 | 0.05487 | 16902.93447 | 0 | Yes | *926.4865 | *927.4943 |

**Table S3.** List of ions detected for $_{200}$CASLQ**K**FGER$_{209}$

| Mass/Charge | Area | Height | Width | Width at 50% | Resolution | Charge | Monoisotopic | Mass(charge) | Mass/charge (charge) |
|---|---|---|---|---|---|---|---|---|---|
| 133.04354 | 0.15624 | 15 | 0.01953 | 0.00929 | 14320.66201 | 0 | Yes | *132.0357 | *133.0435 |
| 141.06564 | 0.09385 | 13 | 0.01676 | 0.00584 | 24173.48263 | 0 | Yes | *140.0578 | *141.0656 |
| 175.11917 | 0.17178 | 14 | 0.02241 | 0.01162 | 15072.90963 | 0 | Yes | *174.1113 | *175.1192 |
| 187.05333 | 0.13894 | 9 | 0.04246 | 0.01168 | 16021.47752 | 0 | Yes | *186.0455 | *187.0533 |
| 204.08007 | 1.09453 | 77 | 0.04031 | 0.01303 | 15659.91292 | 1 | Yes | *203.0722 (1) | *204.0801 (1) |
| 232.07521 | 0.96513 | 57 | 0.03869 | 0.01499 | 15486.72469 | 0 | Yes | *231.0674 | *232.0752 |
| 248.09226 | 0.39115 | 29 | 0.03556 | 0.01131 | 21942.44202 | 0 | Yes | *247.0844 | *248.0923 |
| 301.09729 | 0.89855 | 46 | 0.06366 | 0.01632 | 18446.71425 | 0 | Yes | *300.0895 | *301.0973 |
| 304.16418 | 0.14765 | 10 | 0.02953 | 0.01476 | 20600.49414 | 0 | Yes | *303.1564 | *304.1642 |
| 319.10687 | 1.3989 | 73 | 0.07058 | 0.01721 | 18540.12378 | 0 | Yes | *318.0990 | *319.1069 |
| 361.18279 | 1.019 | 45 | 0.06436 | 0.02099 | 17211.46781 | 0 | Yes | *360.1750 | *361.1828 |
| 387.16936 | 0.19157 | 12 | 0.04442 | 0.01629 | 23770.2292 | 0 | Yes | *386.1615 | *387.1694 |
| 414.18148 | 0.49391 | 20 | 0.05169 | 0.02355 | 17589.58773 | 1 | Yes | *413.1736 (1) | *414.1815 (1) |
| 432.19139 | 0.20533 | 9 | 0.04107 | 0.02141 | 20183.17094 | 0 | Yes | *431.1836 | *432.1914 |
| 508.25128 | 2.22353 | 71 | 0.11452 | 0.02752 | 18471.0791 | 0 | Yes | *507.2435 | *508.2513 |
| 560.2485 | 0.32396 | 13 | 0.05344 | 0.0268 | 20906.57881 | 1 | Yes | *559.2407 (1) | *560.2485 (1) |
| 607.39155 | 0.35818 | 9 | 0.09041 | 0.03941 | 15411.74203 | 0 | Yes | *606.3837 | *607.3916 |
| 671.30184 | 1.09674 | 28 | 0.10967 | 0.03789 | 17718.2535 | 0 | Yes | *670.2940 | *671.3018 |
| 680.30545 | 4.57456 | 116 | 0.19137 | 0.03613 | 18830.5163 | 0 | Yes | *679.2976 | *680.3055 |
| 782.34297 | 0.35914 | 10 | 0.09472 | 0.03881 | 20159.16969 | 1 | Yes | *781.3351 (1) | *782.3430 (1) |
| 800.35577 | 3.99179 | 81 | 0.13572 | 0.04386 | 18249.45758 | 0 | Yes | *799.3479 | *800.3558 |
| 852.35066 | 0.41606 | 10 | 0.11534 | 0.03419 | 24929.04235 | 0 | Yes | *851.3428 | *852.3507 |
| 910.40426 | 0.85148 | 15 | 0.12772 | 0.05393 | 16882.18082 | 1 | Yes | *909.3964 (1) | *910.4043 (1) |
| 911.38825 | 1.10326 | 20 | 0.12779 | 0.04686 | 19450.64863 | 1 | No | 910.3804 (1) | 911.3883 (1) |
| 928.41472 | 6.43174 | 116 | 0.18917 | 0.05107 | 18179.12411 | 0 | Yes | *927.4069 | *928.4147 |
| 999.42048 | 0.33901 | 9 | 0.14274 | 0.02007 | 49789.3411 | 0 | Yes | *998.4126 | *999.4205 |
| 1041.50012 | 3.70208 | 64 | 0.17304 | 0.0545 | 19110.28662 | 0 | Yes | *1040.4923 | *1041.5001 |
| 1110.51852 | 2.00778 | 30 | 0.18808 | 0.06903 | 16086.34676 | 1 | Yes | *1109.5107 (1) | *1110.5185 (1) |
| 1111.50371 | 0.59272 | 11 | 0.14112 | 0.04032 | 27566.26238 | 1 | No | 1110.4959 (1) | 1111.5037 (1) |
| 1128.52962 | 12.08712 | 172 | 0.24648 | 0.06463 | 17461.38954 | 0 | Yes | *1127.5218 | *1128.5296 |
| 1199.56473 | 1.51495 | 25 | 0.12706 | 0.0657 | 18259.29392 | 0 | Yes | *1198.5569 | *1199.5647 |

**Table S4.** List of ions detected for $_{349}$LA**K**TYETTLEK$_{359}$

| Mass/Charge | Area | Height | Width | Width at 50% | Resolution | Charge | Monoisotopic | Mass(charge) | Mass/charge (charge) |
|---|---|---|---|---|---|---|---|---|---|
| 157.13393 | 1.16029 | 106 | 0.03537 | 0.01028 | 15280.26132 | 0 | Yes | *156.1261 | *157.1339 |
| 165.01756 | 0.85372 | 75 | 0.03263 | 0.01013 | 16288.08549 | 0 | Yes | *164.0097 | *165.0176 |
| 185.12855 | 1.00484 | 79.8 | 0.05376 | 0.01198 | 15459.07735 | 0 | Yes | *184.1207 | *185.1285 |
| 248.09215 | 14.45486 | 871 | 0.10223 | 0.01485 | 16702.48719 | 0 | Yes | *247.0843 | *248.0921 |
| 258.14586 | 1.07004 | 60 | 0.06801 | 0.01608 | 16056.44684 | 0 | Yes | *257.1380 | *258.1459 |
| 265.11816 | 1.9896 | 115 | 0.06433 | 0.01432 | 18519.37237 | 0 | Yes | *264.1103 | *265.1182 |
| 276.15553 | 1.57336 | 84 | 0.07034 | 0.01512 | 18261.74633 | 0 | Yes | *275.1477 | *276.1555 |
| 364.15115 | 1.72325 | 88 | 0.09155 | 0.01695 | 21487.15174 | 0 | Yes | *363.1433 | *364.1511 |
| 366.16709 | 1.30681 | 52 | 0.0756 | 0.02239 | 16355.76254 | 0 | Yes | *365.1593 | *366.1671 |
| 376.1506 | 1.46681 | 59 | 0.08757 | 0.02208 | 17036.13469 | 1 | Yes | *375.1428 (1) | *376.1506 (1) |
| 389.24088 | 1.7009 | 65 | 0.06681 | 0.02517 | 15464.43229 | 0 | Yes | *388.2330 | *389.2409 |
| 394.16172 | 2.96381 | 117 | 0.10085 | 0.02234 | 17645.34144 | 0 | Yes | *393.1539 | *394.1617 |
| 477.23424 | 9.77743 | 300 | 0.14796 | 0.02735 | 17447.02916 | 0 | Yes | *476.2264 | *477.2342 |
| 490.28806 | 2.79625 | 89 | 0.09998 | 0.02611 | 18777.64775 | 0 | Yes | *489.2802 | *490.2881 |
| 578.27564 | 3.27432 | 69 | 0.14251 | 0.05702 | 10141.20164 | 0 | Yes | *577.2678 | *578.2756 |
| 591.33519 | 5.37667 | 146 | 0.13038 | 0.03431 | 17234.16819 | 0 | Yes | *590.3274 | *591.3352 |
| 629.80041 | 1.55097 | 46 | 0.12748 | 0.03364 | 18721.91493 | 0 | Yes | *628.7926 | *629.8004 |
| 638.80444 | 6.14107 | 170 | 0.12839 | 0.03287 | 19434.42978 | 0 | Yes | *637.7966 | *638.8044 |
| 674.32144 | 2.05553 | 56 | 0.08794 | 0.03235 | 20846.38229 | 0 | Yes | *673.3136 | *674.3214 |
| 720.37883 | 7.77871 | 172 | 0.16663 | 0.04117 | 17496.35371 | 0 | Yes | *719.3710 | *720.3788 |
| 721.85927 | 3.19202 | 86 | 0.09857 | 0.03342 | 21601.59571 | 0 | Yes | *720.8514 | *721.8593 |
| 730.86253 | 15.47952 | 381 | 0.16021 | 0.03727 | 19611.33812 | 0 | Yes | *729.8547 | *730.8625 |
| 883.43728 | 24.42514 | 464 | 0.2097 | 0.05142 | 17182.24365 | 0 | Yes | *882.4295 | *883.4373 |
| 966.47924 | 3.16709 | 56 | 0.19301 | 0.05571 | 17348.66472 | 0 | Yes | *965.4714 | *966.4792 |
| 984.48764 | 44.60438 | 718 | 0.25678 | 0.05777 | 17040.75356 | 0 | Yes | *983.4798 | *984.4876 |
| 1258.57983 | 3.03348 | 52 | 0.20023 | 0.06638 | 18959.42999 | 0 | Yes | *1257.5720 | *1258.5798 |
| 1276.5886 | 26.96154 | 371 | 0.28232 | 0.07131 | 17902.90394 | 0 | Yes | *1275.5808 | *1276.5886 |
| 1347.63171 | 13.11524 | 186 | 0.30043 | 0.06509 | 20703.24629 | 0 | Yes | *1346.6239 | *1347.6317 |

**Table S5.** List of ions detected for $_{429}$NLG**K**VGSR$_{436}$

| Mass/Charge | Area | Height | Width | Width at 50% | Resolution | Charge | Monoisotopic | Mass(charge) | Mass/charge (charge) |
|---|---|---|---|---|---|---|---|---|---|
| 102.05596 | 0.03814 | 7.11652 | 0.04846 | 0.00488 | 20914.11897 | 0 | Yes | *101.0481 | *102.0560 |
| 120.07821 | 0.05409 | 7.80601 | 0.05257 | 0.00661 | 18168.12681 | 0 | Yes | *119.0704 | *120.0782 |
| 129.10197 | 0.08578 | 8.84024 | 0.06413 | 0.00797 | 16196.21817 | 2 | Yes | *256.1883 (2) | *129.1020 (2) |
| 130.08333 | 0.06293 | 5.69007 | 0.07403 | 0.00939 | 13856.34784 | 0 | Yes | *129.0755 | *130.0833 |
| 136.07485 | 0.07791 | 7.84244 | 0.06255 | 0.00862 | 15787.01938 | 1 | Yes | *135.0670 (1) | *136.0749 (1) |
| 147.11389 | 0.08453 | 10.48203 | 0.06503 | 0.00691 | 21291.61815 | 0 | Yes | *146.1061 | *147.1139 |
| 155.11489 | 0.07233 | 6.37956 | 0.06678 | 0.01076 | 14417.96446 | 0 | Yes | *154.1071 | *155.1149 |
| 177.05564 | 0.06569 | 9.27542 | 0.06759 | 0.00528 | 33517.82637 | 0 | Yes | *176.0478 | *177.0556 |
| 200.14015 | 0.6162 | 48.51666 | 0.08384 | 0.01098 | 18226.47769 | 0 | Yes | *199.1323 | *200.1402 |
| 228.13636 | 0.44736 | 26.56152 | 0.08951 | 0.0183 | 12465.198 | 1 | Yes | *227.1285 (1) | *228.1364 (1) |
| 248.09202 | 0.21039 | 15.47631 | 0.09334 | 0.01029 | 24104.50875 | 0 | Yes | *247.0842 | *248.0920 |
| 267.15978 | 0.08069 | 6.68401 | 0.08303 | 0.01416 | 18868.82569 | 0 | Yes | *266.1519 | *267.1598 |
| 276.17037 | 0.07721 | 7.15278 | 0.08441 | 0.00968 | 28515.36765 | 0 | Yes | *275.1625 | *276.1704 |
| 291.16356 | 0.18333 | 12.54646 | 0.10594 | 0.01415 | 20577.30743 | 0 | Yes | *290.1557 | *291.1636 |
| 294.18289 | 0.09463 | 5.69025 | 0.09681 | 0.01739 | 16915.92659 | 1 | Yes | *293.1751 (1) | *294.1829 (1) |
| 350.13454 | 0.23908 | 12.89524 | 0.11617 | 0.01525 | 22959.12722 | 0 | Yes | *349.1267 | *350.1345 |
| 390.23257 | 0.48758 | 17.59055 | 0.12822 | 0.02756 | 14157.68821 | 0 | Yes | *389.2247 | *390.2326 |
| 391.20207 | 0.16656 | 12.25339 | 0.13954 | 0.00938 | 41691.1794 | 0 | Yes | *390.1942 | *391.2021 |
| 409.54466 | 0.38784 | 16.25075 | 0.13706 | 0.02387 | 17160.52583 | 3 | Yes | *1225.6105 (3) | *409.5447 (3) |
| 409.8781 | 0.23515 | 10.13163 | 0.11998 | 0.02188 | 18729.12273 | 3 | No | 1226.6108 (3) | 409.8781 (3) |
| 410.20852 | 0.08234 | 9.23916 | 0.06859 | 0.00774 | 53023.68704 | 3 | No | 1227.6021 (3) | 410.2085 (3) |
| 426.72779 | 0.11397 | 5.69025 | 0.11659 | 0.02095 | 20373.35875 | 0 | Yes | *425.7200 | *426.7278 |
| 427.18728 | 0.12003 | 6.3433 | 0.12249 | 0.01534 | 27841.54993 | 3 | Yes | *1278.5384 (3) | *427.1873 (3) |
| 466.72409 | 0.31366 | 9.13366 | 0.15242 | 0.03735 | 12495.4894 | 0 | Yes | *465.7163 | *466.7241 |
| 475.23531 | 0.17092 | 5.91114 | 0.11074 | 0.02957 | 16071.96543 | 0 | Yes | *474.2275 | *475.2353 |
| 483.60586 | 0.15509 | 10.48203 | 0.06826 | 0.0137 | 35310.2622 | 0 | Yes | *482.5980 | *483.6059 |
| 483.75364 | 1.29649 | 39.27639 | 0.11793 | 0.02919 | 16573.98214 | 0 | Yes | *482.7458 | *483.7536 |
| 519.26105 | 0.25144 | 7.11798 | 0.13504 | 0.03948 | 13152.80964 | 1 | Yes | *518.2532 (1) | *519.2610 (1) |
| 537.26384 | 0.37692 | 13.92198 | 0.15699 | 0.02292 | 23441.98202 | 1 | Yes | *536.2560 (1) | *537.2638 (1) |
| 540.28214 | 0.16199 | 9.75668 | 0.12463 | 0.01044 | 51747.52459 | 2 | Yes | *1078.5486 (2) | *540.2821 (2) |
| 599.3637 | 0.11374 | 5.69007 | 0.13127 | 0.02015 | 29742.96951 | 1 | Yes | *598.3559 (1) | *599.3637 (1) |
| 613.80338 | 0.33812 | 10.01028 | 0.17479 | 0.03493 | 17571.72751 | 2 | Yes | *1225.5911 (2) | *613.8034 (2) |
| 614.31407 | 0.20871 | 8.3233 | 0.16786 | 0.01689 | 36371.85853 | 2 | No | 1226.6125 (2) | 614.3141 (2) |
| 666.30526 | 0.27733 | 8.4444 | 0.11655 | 0.04175 | 15959.31561 | 0 | Yes | *665.2974 | *666.3053 |
| 682.34742 | 0.19721 | 7.11669 | 0.17692 | 0.03737 | 18258.55757 | 0 | Yes | *681.3396 | *682.3474 |
| 705.349 | 0.10026 | 7.11652 | 0.12741 | 0.01283 | 54981.94365 | 0 | Yes | *704.3412 | *705.3490 |
| 723.33272 | 0.14838 | 7.15295 | 0.15179 | 0.01351 | 53526.28684 | 1 | Yes | *722.3249 (1) | *723.3327 (1) |
| 739.36365 | 5.31381 | 106.45715 | 0.24555 | 0.04788 | 15442.45872 | 0 | Yes | *738.3558 | *739.3637 |
| 852.43659 | 0.27978 | 10.96306 | 0.17303 | 0.01805 | 47228.13062 | 0 | Yes | *851.4288 | *852.4366 |
| 862.43795 | 1.99545 | 37.9659 | 0.25691 | 0.04756 | 18135.37004 | 0 | Yes | *861.4301 | *862.4379 |
| 951.44465 | 0.27766 | 9.32377 | 0.1828 | 0.04308 | 22083.1908 | 1 | Yes | *950.4368 (1) | *951.4446 (1) |

**Table S6.** List of ions detected for $_{525}$**K**QTALVELVK$_{534}$

| Mass/Charge | Area | Height | Width | Width at 50% | Resolution | Charge | Monoisotopic | Mass(charge) | Mass/charge (charge) |
|---|---|---|---|---|---|---|---|---|---|
| 147.11301 | 0.60584 | 54 | 0.02738 | 0.0104 | 14148.09714 | 0 | Yes | *146.1052 | 147.11301 |
| 165.01799 | 1.13829 | 92 | 0.03625 | 0.01111 | 14854.74431 | 0 | Yes | *164.0102 | 165.01799 |
| 246.18096 | 1.17336 | 72 | 0.06199 | 0.01458 | 16884.21078 | 0 | Yes | *245.1731 | 246.18096 |
| 248.0919 | 12.45688 | 784 | 0.11557 | 0.01426 | 17400.56096 | 0 | Yes | *247.0841 | 248.0919 |
| 265.11894 | 1.70701 | 98 | 0.06433 | 0.01536 | 17262.13704 | 0 | Yes | *264.1111 | 265.11894 |
| 310.14015 | 7.8597 | 406 | 0.0994 | 0.01732 | 17902.27254 | 0 | Yes | *309.1323 | 310.14015 |
| 342.20318 | 1.2555 | 64 | 0.06787 | 0.0173 | 19783.87993 | 0 | Yes | *341.1954 | 342.20318 |
| 359.26626 | 1.79455 | 79 | 0.11767 | 0.0188 | 19109.09643 | 0 | Yes | *358.2584 | 359.26626 |
| 404.14654 | 2.40543 | 92 | 0.0851 | 0.02344 | 17241.96099 | 0 | Yes | *403.1387 | 404.14654 |
| 421.17263 | 8.49896 | 311 | 0.09846 | 0.02404 | 17516.27187 | 0 | Yes | *420.1648 | 421.17263 |
| 488.30928 | 3.71665 | 121 | 0.11225 | 0.0277 | 17626.90163 | 0 | Yes | *487.3014 | 488.30928 |
| 496.27727 | 1.67854 | 56 | 0.10059 | 0.02969 | 16716.99639 | 0 | Yes | *495.2694 | 496.27727 |
| 504.21008 | 1.22615 | 49 | 0.06337 | 0.02371 | 21265.87244 | 1 | Yes | *503.2022 (1) | 504.21008 |
| 514.28809 | 1.65113 | 53 | 0.0832 | 0.02934 | 17531.22807 | 0 | Yes | *513.2803 | 514.28809 |
| 522.22106 | 3.63073 | 109 | 0.14833 | 0.02999 | 17414.16532 | 0 | Yes | *521.2132 | 522.22106 |
| 587.37871 | 4.03523 | 102 | 0.13679 | 0.03581 | 16400.45151 | 0 | Yes | *586.3709 | 587.37871 |
| 627.37199 | 2.29016 | 60 | 0.1343 | 0.03534 | 17751.50229 | 0 | Yes | *626.3642 | 627.37199 |
| 629.34363 | 3.84416 | 114 | 0.12743 | 0.03263 | 19284.51213 | 0 | Yes | *628.3358 | 629.34363 |
| 637.85684 | 5.65187 | 167 | 0.10691 | 0.03022 | 21109.66511 | 2 | Yes | *1273.6980 (2) | 637.85684 |
| 638.3492 | 2.55252 | 71 | 0.09982 | 0.03418 | 18673.99521 | 2 | No | 1274.6827 (2) | 638.3492 |
| 646.86185 | 31.71881 | 866.2 | 0.20815 | 0.03397 | 19042.82204 | 0 | Yes | *645.8540 | 646.86185 |
| 700.46207 | 5.93022 | 138 | 0.13444 | 0.03952 | 17723.22558 | 0 | Yes | *699.4542 | 700.46207 |
| 771.50001 | 10.16637 | 219 | 0.2038 | 0.03983 | 19370.71209 | 0 | Yes | *770.4922 | 771.50001 |
| 872.54706 | 41.52942 | 749 | 0.30843 | 0.04964 | 17578.01607 | 0 | Yes | *871.5392 | 872.54706 |
| 983.58016 | 3.69946 | 65 | 0.18586 | 0.05244 | 18756.14167 | 1 | No | 982.5723 (1) | 983.58016 |
| 1000.60773 | 6.73517 | 105 | 0.21424 | 0.06069 | 16486.27723 | 0 | Yes | *999.5999 | 1000.60773 |
| 147.11301 | 0.60584 | 54 | 0.02738 | 0.0104 | 14148.09714 | 0 | Yes | *146.1052 | 147.11301 |
| 165.01799 | 1.13829 | 92 | 0.03625 | 0.01111 | 14854.74431 | 0 | Yes | *164.0102 | 165.01799 |

**Table S7.** List of ions detected for $_{539}$AT**K**EQLK$_{545}$

| Mass/Charge | Area | Height | Width | Width at 50% | Resolution | Charge | Monoisotopic | Mass(charge) | Mass/charge (charge) |
|---|---|---|---|---|---|---|---|---|---|
| 145.0967 | 0.76653 | 69 | 0.03059 | 0.00953 | 15232.62123 | 0 | Yes | *144.0889 | 145.0967 |
| 147.11154 | 0.59214 | 49 | 0.03081 | 0.01007 | 14601.84157 | 0 | Yes | *146.1037 | 147.11154 |
| 173.09161 | 0.8595 | 68 | 0.03341 | 0.01189 | 14551.76868 | 0 | Yes | *172.0838 | 173.09161 |
| 248.09087 | 3.54258 | 208 | 0.07556 | 0.01551 | 15994.44396 | 0 | Yes | *247.0830 | 248.09087 |
| 260.19648 | 0.95593 | 63 | 0.05918 | 0.01317 | 19756.15222 | 0 | Yes | *259.1886 | 260.19648 |
| 388.25478 | 1.16771 | 55 | 0.09453 | 0.01959 | 19814.78257 | 0 | Yes | *387.2470 | 388.25478 |
| 394.15852 | 1.09251 | 45 | 0.10645 | 0.02056 | 19175.55258 | 0 | Yes | *393.1507 | 394.15852 |
| 405.20558 | 1.21565 | 56 | 0.06817 | 0.01976 | 20503.26894 | 0 | Yes | *404.1978 | 405.20558 |
| 422.15433 | 1.04657 | 41 | 0.06958 | 0.02411 | 17510.77151 | 0 | Yes | *421.1465 | 422.15433 |
| 447.18679 | 2.30947 | 78 | 0.11935 | 0.02493 | 17935.07903 | 0 | Yes | *446.1790 | 447.18679 |
| 455.73 | 3.72908 | 140 | 0.12049 | 0.02277 | 20015.82437 | 0 | Yes | *454.7222 | 455.73 |
| 482.24381 | 6.0484 | 218 | 0.13014 | 0.02709 | 17799.87665 | 0 | Yes | *481.2360 | 482.24381 |
| 491.24877 | 19.87122 | 678 | 0.1376 | 0.02656 | 18495.00179 | 0 | Yes | *490.2409 | 491.24877 |
| 499.28698 | 1.43454 | 43 | 0.1072 | 0.03018 | 16546.1002 | 1 | Yes | *498.2791 (1) | 499.28698 |
| 517.29577 | 6.82596 | 240 | 0.15404 | 0.02566 | 20157.50228 | 0 | Yes | *516.2879 | 517.29577 |
| 550.21116 | 1.57543 | 46 | 0.10591 | 0.02606 | 21110.00955 | 0 | Yes | *549.2033 | 550.21116 |
| 809.40151 | 25.90019 | 495 | 0.21677 | 0.05043 | 16049.69837 | 0 | Yes | *808.3937 | 809.40151 |
| 892.44219 | 1.28984 | 36 | 0.10959 | 0.03398 | 26266.08966 | 0 | Yes | *891.4344 | 892.44219 |
| 910.45042 | 7.85083 | 142 | 0.17881 | 0.05496 | 16564.65681 | 0 | Yes | *909.4426 | 910.45042 |
| 920.43421 | 2.15752 | 38 | 0.12842 | 0.0585 | 15732.84321 | 0 | Yes | *919.4264 | 920.43421 |

**Table S8.** List of ions detected for ${}_{542}$EQL**K**AVMDDFAAFVEK${}_{557}$

| Mass/Charge | Area | Height | Width | Width at 50% | Resolution | Charge | Monoisotopic | Mass(charge) | Mass/charge (charge) |
|---|---|---|---|---|---|---|---|---|---|
| 129.06547 | 0.36869 | 38 | 0.02244 | 0.0087 | 14829.47043 | 0 | Yes | *128.0576 | *129.0655 |
| 165.01824 | 0.6779 | 55 | 0.03625 | 0.01141 | 14459.14734 | 0 | Yes | *164.0104 | *165.0182 |
| 240.09813 | 2.29349 | 144 | 0.0481 | 0.01466 | 16376.14994 | 0 | Yes | *239.0903 | *240.0981 |
| 241.08198 | 0.62658 | 33 | 0.07887 | 0.01587 | 15189.76843 | 1 | Yes | *240.0742 (1) | *241.0820 (1) |
| 248.09171 | 10.19662 | 591 | 0.07556 | 0.01575 | 15754.58453 | 0 | Yes | *247.0839 | *248.0917 |
| 258.10834 | 3.42978 | 205 | 0.05894 | 0.01522 | 16953.90712 | 0 | Yes | *257.1005 | *258.1083 |
| 258.14407 | 0.91362 | 61 | 0.04081 | 0.01527 | 16905.95181 | 0 | Yes | *257.1362 | *258.1441 |
| 265.11849 | 0.65248 | 39 | 0.05974 | 0.01407 | 18840.29509 | 0 | Yes | *264.1107 | *265.1185 |
| 276.15558 | 1.13957 | 61 | 0.05159 | 0.01706 | 16182.72846 | 0 | Yes | *275.1477 | *276.1556 |
| 290.15001 | 0.58645 | 30 | 0.07691 | 0.0172 | 16870.34778 | 0 | Yes | *289.1422 | *290.1500 |
| 325.18761 | 0.75316 | 39 | 0.05598 | 0.0173 | 18794.54618 | 1 | Yes | *324.1798 (1) | *325.1876 (1) |
| 334.13997 | 0.57259 | 30 | 0.06706 | 0.01782 | 18750.65874 | 0 | Yes | *333.1321 | *334.1400 |
| 336.15547 | 0.95719 | 50 | 0.06209 | 0.01791 | 18769.14956 | 0 | Yes | *335.1476 | *336.1555 |
| 353.18237 | 2.42897 | 109 | 0.07955 | 0.01927 | 18327.0112 | 1 | Yes | *352.1745 (1) | *353.1824 (1) |
| 362.10218 | 0.71958 | 34 | 0.06981 | 0.01805 | 20062.10749 | 0 | Yes | *361.0943 | *362.1022 |
| 364.1506 | 1.14704 | 50 | 0.07001 | 0.02116 | 17205.80931 | 1 | Yes | *363.1428 (1) | *364.1506 (1) |
| 370.20875 | 1.95471 | 88 | 0.09773 | 0.01925 | 19230.69749 | 1 | Yes | *369.2009 (1) | *370.2087 (1) |
| 371.19327 | 1.54682 | 65 | 0.08699 | 0.02137 | 17371.18341 | 1 | No | 370.1854 (1) | 371.1933 (1) |
| 375.22431 | 1.90778 | 82 | 0.07653 | 0.0207 | 18124.23256 | 0 | Yes | *374.2165 | *375.2243 |
| 406.19708 | 2.58215 | 95 | 0.10806 | 0.02427 | 16736.63665 | 1 | Yes | *405.1893 (1) | *406.1971 (1) |
| 449.16722 | 1.09748 | 41 | 0.10168 | 0.02313 | 19422.76772 | 1 | Yes | *448.1594 (1) | *449.1672 (1) |
| 463.21854 | 1.33316 | 43 | 0.12755 | 0.02605 | 17782.46348 | 0 | Yes | *462.2107 | *463.2185 |
| 477.23464 | 2.47827 | 78 | 0.14179 | 0.02866 | 16650.66836 | 1 | Yes | *476.2268 (1) | *477.2346 (1) |
| 517.22877 | 1.34136 | 38 | 0.1091 | 0.03129 | 16531.40842 | 1 | Yes | *516.2209 (1) | *517.2288 (1) |
| 520.20439 | 1.59624 | 44 | 0.12873 | 0.03043 | 17096.99838 | 1 | Yes | *519.1966 (1) | *520.2044 (1) |
| 522.29251 | 4.4984 | 133 | 0.14833 | 0.03071 | 17007.6214 | 1 | Yes | *521.2847 (1) | *522.2925 (1) |
| 593.32983 | 3.78753 | 104 | 0.15123 | 0.03246 | 18278.69588 | 0 | Yes | *592.3220 | *593.3298 |
| 645.28812 | 5.82806 | 142 | 0.17205 | 0.03797 | 16993.76216 | 1 | Yes | *644.2803 (1) | *645.2881 (1) |
| 651.24699 | 2.26853 | 39 | 0.21606 | 0.04226 | 15410.3624 | 1 | Yes | *650.2392 (1) | *651.2470 (1) |
| 663.29917 | 1.76611 | 40 | 0.14536 | 0.04023 | 16486.35051 | 0 | Yes | *662.2913 | *663.2992 |
| 664.36687 | 2.61856 | 62 | 0.21093 | 0.03546 | 18735.81696 | 1 | Yes | *663.3590 (1) | *664.3669 (1) |
| 716.32529 | 3.19109 | 79 | 0.11329 | 0.03711 | 19304.24519 | 1 | Yes | *715.3175 (1) | *716.3253 (1) |
| 734.33751 | 2.03417 | 45 | 0.13 | 0.04079 | 18004.97521 | 1 | Yes | *733.3297 (1) | *734.3375 (1) |
| 750.31229 | 1.70832 | 39 | 0.1546 | 0.03981 | 18847.63465 | 1 | Yes | *749.3045 (1) | *750.3123 (1) |
| 811.43462 | 2.92607 | 59 | 0.16881 | 0.04604 | 17624.71967 | 1 | Yes | *810.4268 (1) | *811.4346 (1) |
| 833.40363 | 1.75563 | 36 | 0.13849 | 0.04592 | 18149.79498 | 1 | Yes | *832.3958 (1) | *833.4036 (1) |
| 897.38068 | 1.67383 | 32 | 0.14371 | 0.04903 | 18302.21633 | 0 | Yes | *896.3728 | *897.3807 |
| 926.46218 | 3.19531 | 57 | 0.20615 | 0.04993 | 18556.41439 | 0 | Yes | *925.4543 | *926.4622 |
| 1041.48855 | 7.222 | 113 | 0.20946 | 0.05794 | 17974.211 | 0 | Yes | *1040.4807 | *1041.4885 |
| 1172.5294 | 20.72746 | 284 | 0.34787 | 0.06645 | 17644.63471 | 0 | Yes | *1171.5216 | *1172.5294 |
| 1173.53291 | 2.67784 | 36 | 0.23202 | 0.06815 | 17218.75772 | 0 | Yes | *1172.5251 | *1173.5329 |
| 1271.59781 | 16.08084 | 209 | 0.3019 | 0.07049 | 18038.14244 | 0 | Yes | *1270.5900 | *1271.5978 |
| 1272.60276 | 2.26509 | 32 | 0.20134 | 0.07047 | 18058.92186 | 0 | Yes | *1271.5949 | *1272.6028 |
| 1342.63133 | 25.54068 | 325 | 0.35158 | 0.0737 | 18218.68666 | 0 | Yes | *1341.6235 | *1342.6313 |
| 1343.63631 | 3.63082 | 45 | 0.20689 | 0.07343 | 18299.32938 | 0 | Yes | *1342.6285 | *1343.6363 |

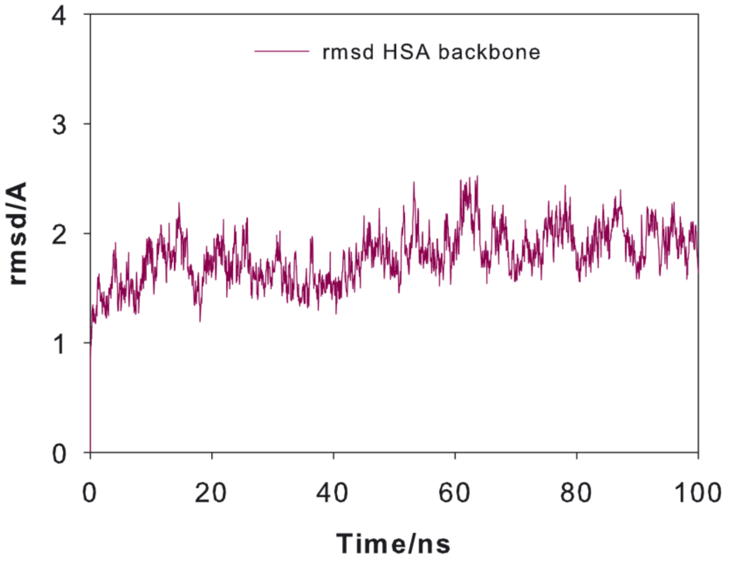


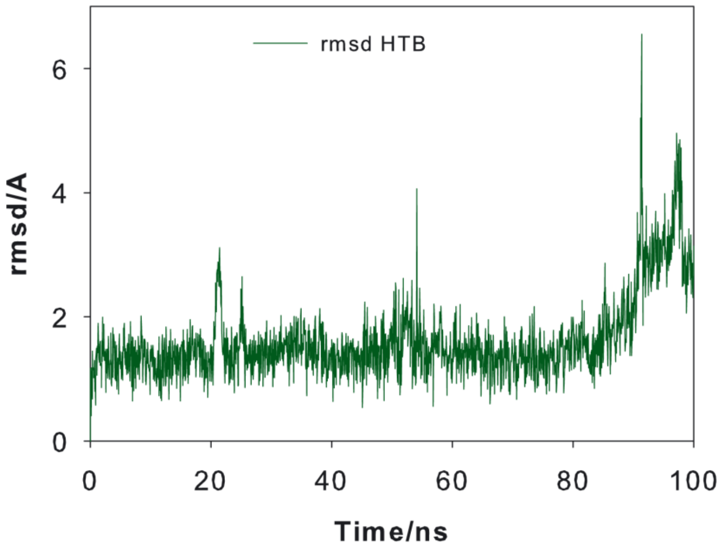


**Figure S16.** The rmsd plots for the protein backbone (Cα, C, N and O atoms) (up) and ligand (down) calculated for the binary HTB@HSA complex obtained from MD simulation studies.
